# A Proteome-Level Investigation Into *Plasmodiophora brassicae* Resistance in *Brassica napus* Canola

**DOI:** 10.3389/fpls.2022.860393

**Published:** 2022-03-24

**Authors:** Dinesh Adhikary, Devang Mehta, R. Glen Uhrig, Habibur Rahman, Nat N. V. Kav

**Affiliations:** ^1^Department of Agricultural, Food and Nutritional Science, University of Alberta, Edmonton, AB, Canada; ^2^Department of Biological Sciences, University of Alberta, Edmonton, AB, Canada

**Keywords:** *Brassica napus*, clubroot, proteomics, calcium binding, plant–pathogen interaction

## Abstract

Clubroot of *Brassicaceae*, an economically important soil borne disease, is caused by *Plasmodiophora brassicae* Woronin, an obligate, biotrophic protist. This disease poses a serious threat to canola and related crops in Canada and around the globe causing significant losses. The pathogen is continuously evolving and new pathotypes are emerging, which necessitates the development of novel resistant canola cultivars to manage the disease. Proteins play a crucial role in many biological functions and the identification of differentially abundant proteins (DAP) using proteomics is a suitable approach to understand plant–pathogen interactions to assist in the development of gene specific markers for developing clubroot resistant (CR) cultivars. In this study, *P. brassicae* pathotype 3 (P3H) was used to challenge CR and clubroot susceptible (CS) canola lines. Root samples were collected at three distinct stages of pathogenesis, 7−, 14−, and 21-days post inoculation (DPI), protein samples were isolated, digested with trypsin and subjected to liquid chromatography with tandem mass spectrometry (LC-MS/MS) analysis. A total of 937 proteins demonstrated a significant (*q*-value < 0.05) change in abundance in at least in one of the time points when compared between control and inoculated CR-parent, CR-progeny, CS-parent, CS-progeny and 784 proteins were significantly (*q* < 0.05) changed in abundance in at least in one of the time points when compared between the inoculated- CR and CS root proteomes of parent and progeny across the three time points tested. Functional annotation of differentially abundant proteins (DAPs) revealed several proteins related to calcium dependent signaling pathways. In addition, proteins related to reactive oxygen species (ROS) biochemistry, dehydrins, lignin, thaumatin, and phytohormones were identified. Among the DAPs, 73 putative proteins orthologous to CR proteins and quantitative trait loci (QTL) associated with eight CR loci in different chromosomes including chromosomes A3 and A8 were identified. Proteins including BnaA02T0335400WE, BnaA03T0374600WE, BnaA03T0262200WE, and BnaA03T0464700WE are orthologous to identified CR loci with possible roles in mediating clubroot responses. In conclusion, these results have contributed to an improved understanding of the mechanisms involved in mediating response to *P. brassicae* in canola at the protein level.

## Introduction

Canola (*Brassica napus* L.) is a widely cultivated, cruciferous oilseed crop. Globally, 68.87 million metric tons of canola was produced in 2020, making it an important agricultural commodity (Shahbandeh, 2021). Since its development ∼50 years ago, Canada has led the world in canola production (Canola Council of Canada, 2021 – Industry overview), generating $29.9 billion annually in economic activities (Canola Council of Canada, 2021 – Industry Overview). For over a century, *Brassica* crop yield has been negatively affected by *P. brassicae* Woronin, which causes clubroot disease (Cook and Schwartz, 1930; Buczacki, 1983). This disease has impacted > 56 countries in all of Asia, Europe, Americas, some parts of Oceania and Africa, leading to yield losses of ∼15% globally (Dixon, 2009; Czubatka-Bieńkowska et al., 2020). Development of CR canola cultivars has been successful in combating the disease but the evolution of new pathotypes of *P. brassicae* that can break down the resistance continues to be a major problem (Strelkov et al., 2018).

*Plasmodiophora brassicae*, the soil-borne, obligate, biotrophic protist pathogen produces resting spores, which can survive in soil for many years and, when the conditions are favorable, serve as the inoculum to infect susceptible plants resulting in the death of ∼50% of the infected plants (Gibbs, 1931; Wallenhammar, 1996; Howard et al., 2010; Hwang et al., 2012). Clubroot development in *Brassica* spp. is favored by warm temperatures (20–25°C) and wet soils with relatively lower soil pH (<6.5; Dixon, 2009; Gossen et al., 2013). Disease progression in roots is characterized by a primary plasmodia stage marked by the infection of root hairs, followed by secondary plasmodia phase indicated by cortical cell infection, which escalates into increased and irregular vascular cambium activity, reduction in xylem tissue, increase in parenchyma cells, and eventually the deformity of roots (Kageyama and Asano, 2009; Hwang et al., 2012; Malinowski et al., 2012). Pathogen progression in root tissues trigger phytohormone (auxin and cytokinin) imbalance leading to hypertrophy and hyperplasia, which results in the formation of clubs in the roots (Siemens et al., 2006; Ludwig-Müller et al., 2009, 2017; Jahn et al., 2013). Once the pathogen advances to the secondary plasmodia phase, significant damage on the root tissues occur and infection sites become physiological sinks, leading to the disruption of water and mineral conduction, resulting in stunting, wilting and eventual death of plants (Keen and Williams, 1969; Walerowski et al., 2018).

In different parts of the world, clubroot management programs involve the application of soil amendments (e.g., lime) or chemical fungicides, crop rotation, and field equipment sanitization; however, many of these conventional methods are either too expensive or ineffective in large farm settings (Donald and Porter, 2009). As alluded to earlier, recently, growers have depended heavily on CR cultivars; however, the emergence of novel *P. brassicae* pathotypes and their rapid spread have posed serious threats to the management of clubroot disease in canola (Strelkov et al., 2018; Hollman et al., 2021). Numerous studies have been conducted to understand the biology and races of *P. brassicae* (Williams, 1966; Kageyama and Asano, 2009), and to identify quantitative trait loci (QTL) related to the disease resistance in *Brassica* species (Nagaoka et al., 2010; Hasan et al., 2021). To date approximately 24 QTL and over 400 putative clubroot resistance genes have been reported and mapped to the A and C genomes of different *Brassica* spp. (Nagaoka et al., 2010; Dakouri et al., 2021; Hasan et al., 2021). Among these, multiple QTL were mapped to each of chromosomes A3 and A8 in *B. rapa.* Although many of the putative clubroot resistance related genes are yet to be confirmed, their putative physical location has been reported by different researchers (Yu et al., 2017; Hasan et al., 2021).

Advances in next generation sequencing (NGS) and other “omics”-based approaches have contributed to the identification of many putative genes involved in mediating responses in *Brassica* spp. to the clubroot pathogen. For instance, RNA sequencing studies in *B. oleracea*, *B. rapa*, *B. napus*, *Arabidopsis thaliana* have implicated a role for hormonal signaling in response to the pathogen and profiled several putative clubroot resistance related gene transcripts and lncRNAs (Chen et al., 2016; Zhang et al., 2016; Zhao et al., 2017; Summanwar et al., 2019, 2020, 2021; Galindo-González et al., 2020). Similarly, there are a few reports of metabolomics-based approaches in *Brassica* spp. that have documented metabolome-level changes in response to the pathogen including a potential role for gluconasturtiin in conferring resistance against the pathogen (Pedras et al., 2008; Wagner et al., 2012, 2019). Proteome-level changes in canola in response to the clubroot pathogen have also been described, which included characterization of root proteomes in response to infection in susceptible canola (Cao et al., 2008). Ji et al. (2018) reported DAPs during early stages of infection and suggested a role for phytohormone regulation during the host-pathogen interaction. Song et al. (2016) characterized the proteome-level changes in *B. rapa* genotypes with and without clubroot resistance gene (*Rcr1*) and reported 527 DAPs, which could be potentially involved in mediating clubroot resistance. Su et al. (2018) and Lan et al. (2019) have also identified DAPs during the secondary stage of infection in CR and CS genotypes of *B. rapa*. Their studies suggested that the proteins involved in brassinosteroids (BR) and cytokinin (CK) pathways were potentially involved in the regulation of clubroot development. Due to the important role played by proteins in mediating various cellular processes, including host–pathogen interactions, the identification and characterization of DAPs may be a viable approach to identify the proteins potentially involved in mediating resistance to the pathogen (Quirino et al., 2010; Song et al., 2016; Ji et al., 2018; Lan et al., 2019).

In the current study, a comprehensive proteomics analysis at three different stages of infection in CR and CS canola representing CR/CS parent and CR/CS doubled-haploid (DH) progeny lines was conducted and DAPs potentially involved in mediating clubroot resistance were identified. Our results have significance in not only further understanding the molecular mechanisms of clubroot resistance but also in the potential development of protein-specific markers to assist in the selection of CR lines.

## Materials and Methods

### Plant Material

In this study, a set of doubled haploid (DH) lines and their parents were used; detailed information on these lines are available in Hasan and Rahman (2016). Briefly, the DH lines were derived from F_1_ plants of Rutabaga Brookfield-9005 P1 x A07-29NI and A07-29NI x Rutabaga Brookfield-9005 P1 crosses. The parent Rutabaga Brookefield-9005 is a CR inbred line developed from rutabaga (*Brassica napus* var. *napobrassica*) cv. Brookefield-9005 through self-pollination (Hasan and Rahman, 2016) and showed complete resistance to Canadian *P. brassicae* pathotypes 2, 3, 5, 6, and 8. The CS parent used in this study was A07-29NI, which is an open pollinated spring *B. napus* canola cultivar grown in Canada as UA AlfaGold (Rahman, 2016).

### Preparation of *Plasmodiophora brassicae* Inoculum, Infection of Plants and Disease Assessment

A single spore isolate of *P. brassicae*, SACAN-ss1 belonging to pathotype 3 (P3H) was used to challenge the seedlings. Resting spores of the pathogen were isolated essentially as described by Williams (1966). Briefly, infected root tissues with visible clubroot galls (rated disease score of 3/3) were collected, washed, and stored at –80°Celsius until use. Frozen gall tissues (38 g) were thawed and homogenized in sterile deionized water (400 ml) using a mechanical blender. Homogenized tissue was filtered through eightfold layers of cheesecloth (American Fiber & Finishing Inc., Albemarle, NC, United States) and the density of the resting spores in the filtrate was determined using a hemocytometer (VWR, Mississauga, ON, Canada). Final resting spore density was adjusted to 1 × 10^7^ spores/ml with sterilized deionized water (600 ml).

The resistance and susceptibility of all 11 CR and 9 CS DH lines used in this study was confirmed prior to experimentation. All experimental plants were grown in a greenhouse maintaining 400 μmol/m^2^ of light intensity at 22/15°C (light/dark) with a 16/8 h photoperiod in cells filled with Sunshine Professional Growing Mix (Sunshine Horticulture, Bellevue, NE, United States). When seedlings were at 2–3 true leaf stage they were directly injected, close to the root, with the spore suspension (1 ml of 1 × 10^7^ spores/ml). Control plants were treated with sterile water and maintained in separate trays under identical growth conditions. The soil was saturated with water for the first week following inoculation and subsequently watering was performed as required to keep the soil moist. Mixed fertilizer, Nitrogen-Phosphorous-Potassium (20-20-20) was applied once in a week.

Disease screening was performed at 45 DPI as follows: roots were washed thoroughly with tap water, and symptoms were rated visually on a 0–3 scale as described by Kuginuki et al. (1999), where 0 = no visible symptom of clubroot galls, 1 = a small gall mainly on lateral roots, 2 = small galls on the main and lateral roots, and 3 = severe clubbing or the entire root system was deformed. The disease index (DI) was calculated as follows: DI = [(n_1_ + 2n_2_ + …3n_n_)]/(N_T_x3)] × 100, where n_1_, n_2 …_ n_n_ indicate the individuals from a line with the level of disease severity (0–3) and N_T_ indicates the total number of individuals in the line (Li et al., 2016).

### Confirmation of Infection Using Microscopy

To assess the changes in the root tissues, histology and scanning electron microscopy (SEM) of the infected and uninfected tissues at 7−, 14−, and 21− DPI were performed. For histology, samples were prepared as described by Summanwar et al. (2019). Stained tissue sections were dried at 37°C and visualized with a Leica DMRXA light microscope (Meyer Instruments, Houston, TX, United States). Images were acquired with a QI Click digital camera and processed using Q Capture Pro 7 software (Q Imaging, Surrey, BC, Canada).

For SEM, all steps until tissue sectioning were followed according to Summanwar et al. (2019). Tissue sections were collected on SEM pin stubs, air dried, and dewaxed in toluene twice for 5 min and then washed with 100% ethanol twice for 2 min. Samples were further air dried and subjected to sputter coating with palladium (Pd) for 120 seconds using Hummer 6.2 Sputter Coater (Anatech Ltd, Battle Creek, MI, United States). Once coated, tissues were subjected to high resolution electron imaging using ZEISS EVO 10 Scanning Electron Microscope (SEM) (Carl Zeiss Canada Ltd, Toronto, ON, Canada) (White Plains, NY, United States).

### Sample Collection for Protein Extraction

Tissue samples (72 in total) comprised of four genotypes [CR-parent (10 individuals pooled per biological replicate), CS-parent (10 individuals pooled per biological replicate), CR-progeny-bulk (20 individuals pooled per biological replicate representing 10 lines), and CS-progeny-bulk (16 individuals pooled per biological replicate representing 8 lines)]. Root samples were collected at three different times points, 7−, 14−, and 21-DPI as per our previous studies (Summanwar et al., 2019). Three independent experiments comprised of three biological replicates in each experiment and two treatments (inoculated and control) made up the 72 samples (4 genotypes × 3 independent experiments × 3 biological replicates × 2 treatments = 72).

### Extraction of Protein for Proteome Analysis

Frozen root tissue samples described above were homogenized in liquid nitrogen using a mortar and pestle. Homogenized tissue (200 mg) was used for protein extraction using a 1:2 (w/v) ratio of 50 mM HEPES-KOH pH 8.0. 50 mM NaCl, and 4% SDS. Samples were solubilized by vortexing and incubated at 95°C with agitation at 600 rpm for 15 min. After cooling to room temperature, samples were centrifuged at 20,000 *g* for 5 min and the supernatants were transferred to fresh Eppendorf microcentrifuge tubes (2.0 ml). Protein estimation was then performed using a bicinchonic acid (BCA) assay (23225; ThermoScientific, Mississauga, ON, Canada) and samples were normalized to 100 μg each in a total volume of 250 μL. Samples were then reduced with 10 mM dithiothreitol (DTT) at 95°C for 5 min, cooled to room temperature and alkylated with 30 mM iodoacetamide for 30 min in the dark and quenched with 10 mM DTT.

Proteins were digested using a magnetic bead based protocol using a 1:1 mix of hydrophilic and hydrophobic Sera-Mag SpeedBead Carboxylate-modified magnetic particles (45152105050250 and 65152105050250; GE Life Sciences, Boston, MA, United States) (Leutert et al., 2019). Tryptic hydrolysis of protein samples was performed overnight at 37°C using sequencing-grade trypsin (V5113; Promega, Madison, WI, United States). Digested peptides were quantified using a Nanodrop and acidified by adding formic acid (FA) to a final concentration of 5%. The peptide samples were then dried by vacuum centrifugation and dissolved in 3% acetonitrile (ACN) containing 0.1% trifluoroaceticacid (TFA) and desalted using ZipTip C18 pipette tips (ZTC18S960; Millipore, Oakville, ON, Canada). Desalting was performed by pre-equilibrating each tip sequentially with 100% ACN, 60% ACN/0.1% TFA, and 3% ACN/0.1% TFA. Samples were adsorbed by pipetting ten times followed by three washes with 3% ACN/0.1% TFA and eluted with 10 μL of 60% ACN/0.1% TFA five times. Desalted peptides were dried and dissolved in 3.0% ACN/0.1% FA prior to liquid chromatography with tandem mass spectrometry (LC-MS/MS) analysis.

### Nanoflow Liquid Chromatography With tandem Mass Spectrometry Analysis

Peptides were analyzed using a Fusion Lumos Tribrid Orbitrap mass-spectrometer (ThermoFisher Scientific) using a BoxCarDIA data acquisition scheme as described previously (Mehta et al., 2022). Briefly, the dissolved peptides were injected using an Easy-nLC 120 system (LC140; ThermoFisher Scientific) and separated using a 50 cm Easy-Spray PepMap C18 column (ES803A; ThermoFisher Scientific). The column was equilibrated with 100% solvent A (0.1% FA) and the samples were eluted using a segmented solvent B gradient (80% ACN/0.1% FA) from 4 to 41% B (0–65 min). MS^1^ acquisition was performed using two multiplexed tSIM scans of 10 BoxCar windows each spanning a range of 350–1400 m/z. Detection was performed at 120,000 resolution and normalized AGC targets of 100% per isolation window. MS^2^ acquisition was performed using twenty-eight 38.5 m/z windows with an overlap of 1 m/z and a minimum m/z of 200. Resolution was set to 30,000 using a dynamic maximum injection time and the minimum desired points across each peak was set to 6.

### Data Processing and Bioinformatics Analysis

BoxCarDIA raw files were processed using Spectronaut 14 (Biognosys AG, Wagistrasse, Schlieren Switzerland) under a directDIA analysis mode and default search parameters without *N*-acetyl variable modification as described in Mehta et al. (2022). Spectra were searched using a published *B. napus* Westar proteome (Song et al., 2020). Trypsin specificity was set to two missed cleavages and a protein and PSM false discovery rate of 1% each. Data filtering criteria was set to *q*-value ≤ 0.01 and global normalization with MS2-level quantification was performed. Comparative analysis between samples was performed using Spectronaut with a significance threshold of FDR-corrected *p*-value (Dunn, 1961) to identify differentially abundant proteins.

### Functional Classification and Annotation of Differentially Abundant Proteins

Proteome analysis was carried out following standard method similar to published works (Kanehisa et al., 2016; Mistry et al., 2021) predicted proteins were annotated using the gene descriptions from KEGG, Pfam, and Uniprot databases to gain additional insights into their biological roles. TAIR hit IDs corresponding to proteins with significant differentially expressed values (*q*-value < 0.05, after Dunn, 1961) were taken for gene ontology (GO) enrichment analysis using AgriGo (Du et al., 2010; Tian et al., 2017). *Arabidopsis* gene model (TAIR9) was used in the background and the following parameters were applied to run the analysis – statistical test method (Hypergeometric), multi-test adjustment statistical method [Yekutieli (FDR under dependency)], significance level (0.05), minimum number of mapping entries (5), and gene ontology type (complete GO).

## Results

### Disease Progression Following *Plasmodiophora brassicae* Challenge

We compared the changes occurring in the roots of CR and CS lines following the pathogen infection at three different time points using light and scanning and electron microscopy (SEM). The results obtained at each time points are described below.

#### 7 Days Post Inoculation

**FIGURE 1:**
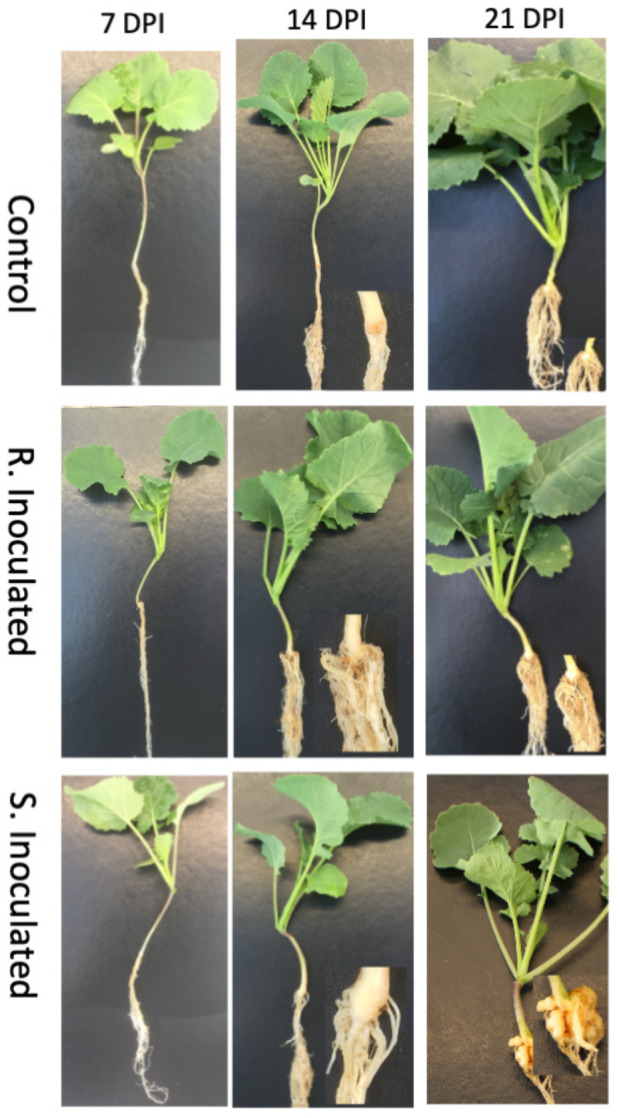
Clubroot gall development following inoculation with *P. brassicae* pathotype 3. Control at 7-, 14-, and 21-DPI, Clubroot resistant (CR) inoculated line at 7-, 14-, and 21-DPI Clubroot susceptible (CS) inoculated 7-, 14-, and 21-DPI.

**FIGURE 2:**
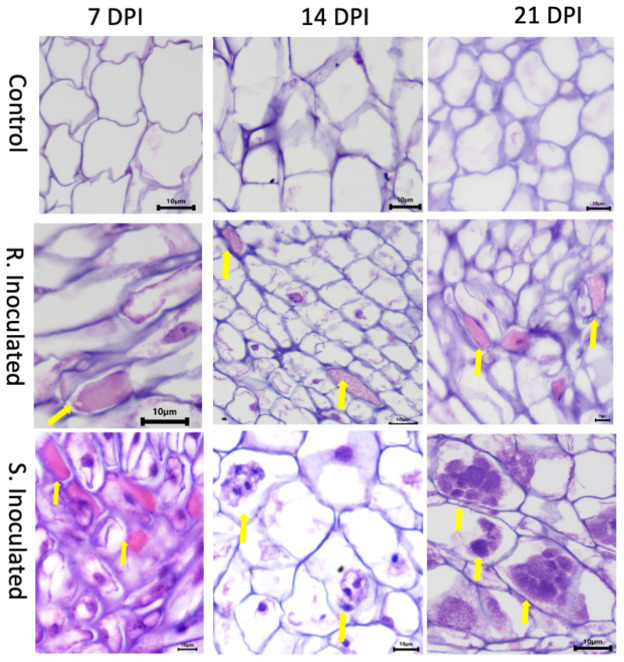
Histology images of root cross sections after *P. brassicae* infection at 7-, 14-, and 21-days post inoculation (DPI). Root tissues were stained with eosin and hematoxylin. Each column indicates DPI of the pathogen and the rows show the control and inoculated genotypes [clubroot- susceptible (CS) and resistant (CR) lines]. At 7 DPI, infected cells showed primary plasmodia with dark purple mass within cells indicated by the solid yellow arrow. At 14 DPI, CS inoculated line showed the presence of secondary plasmodia; however, the pathogen development on the CR inoculated line was not progressed to secondary plasmodia phase. At 21 DPI, pathogen clearly progressed to secondary plasmodia phase, maturing into developing resting spores in the CS line. However, the infection development was not progressed further at the same time point on the CR line.

**FIGURE 3:**
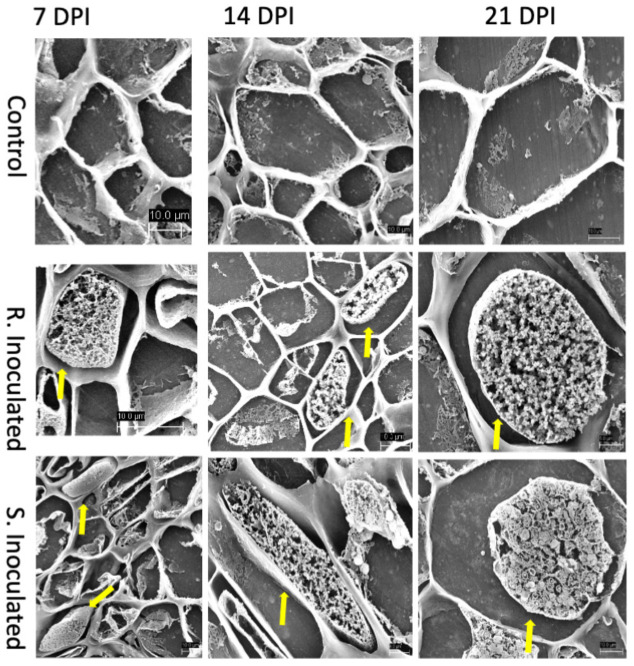
Scanning electron micrograph (SEM) of root cross sections after *P. brassicae* infection at 7-, 14-, and 21-DPI. Each column indicates days after inoculation of the pathogen and the rows show the control and inoculated genotypes (CR and CS). At 7 DPI, infected cells showed primary plasmodia within cells indicated by the solid yellow arrow. At 14 DPI, CS inoculated line showed the presence of secondary plasmodia; however, the pathogen development on the CR inoculated line was not progressed to secondary plasmodia phase. At 21 DPI, pathogen clearly progressed to secondary plasmodia phase, maturing into resting spores. However, the infection development was not progressed in the CR line at the timepoint.

At this stage of infection, the morphology of plant roots and shoots were similar in both control and inoculated groups, in both CR and CS lines with roots being free of galls (Figure 1). Microscopic examination of root tissues indicated that *P. brassicae* was present in the early developmental stage in the vegetative plasmodia phase, colonized around the root hairs, penetrated the cell wall barrier, and established in the root system (Figures 2, 3). Infected cells in both CR and CS lines appeared to be similar in histology sections and appeared as a mass of pink matrices at the infected sites (Figure 2) and under SEM, cells appeared as a tight mass of pathogen cells, which could be distinguished from the uninfected cells (Figure 3).

#### 14 Days Post Inoculation

At 14 DPI, we observed that the root tissues and the cortical region of roots was also penetrated by the pathogen forming mature secondary plasmodia, and larger areas of the root tissue were found to be infected (Figures 2, 3). Histology sections showed that the disease progression was apparent in the CS lines, with a condensed mass of cells arranged at the center (Figure 2). However, in the CR lines, the pink matrix had condensed and loosened giving a granular pink dot like appearance indicating a slower progression of the pathogen (Figure 2). Similarly, under SEM, loosened granular materials were observed in the infected cells in the CR and CS line (Figure 3).

#### 21 Days Post Inoculation

At 21 DPI, not only the clubroot galls were clearly visible on the CS roots (Figure 1), but also the plasmodia, sporangia, and resting spores of the pathogen could also be clearly visualized in under light microscopy as well as SEM, indicating disease progression and infection of the root cortical region (Figures 2, 3). The color of pathogen cells changed to dark purple with multiple clusters of maturing secondary plasmodium (Figure 2) and SEM micrographs indicated maturing secondary plasmodium in a cluster of pathogen cells within the infected host tissues (Figure 3). The whole plant roots appeared swollen with clubs and deformed the root architecture (Figure 1). However, in the CR roots, disease progression was slow and appearance of infected cells was similar at 14 and 21 DPI, showing pink matrix in the cells under histology sections and loosely packed granular pathogen in the infected cells under SEM.

#### 45 Days Post Inoculation

At 45 DPI, 98% of the CR lines did not develop any galls and there were clear visual differences between the roots of CR and CS plants (Supplementary Figure 1). All inoculated CS and CR lines had a disease severity index (DSI) rating > 98% and <5%, respectively (Supplementary Table 1). We selected 7-, 14-, and 21-DPI for our proteome studies since these stages appear to be important for disease progression as evidenced by our microscopic studies and correspond with our previous transcriptome-level studies (Summanwar et al., 2019).

### Identification of Differentially Abundant Proteins

Protein profiles from control and inoculated CR and CS root samples, from both progeny and parent lines were analyzed. We detected an average of 3281 proteins across all the genotypes, treatments, and timepoints, with a low of 2296 proteins in the 14 DPI control CR-parent lines and a high of 3689 proteins at 21 DPI control CS-progeny lines (Table 1). Among these proteins, 937 were significantly (*q* < 0.05) differentially abundant in at least in one of the time points when compared between control and inoculated root proteomes of CR-parent, CR-progeny, CS-parent, CS-progeny across the three time points (Supplementary Table 2). Similarly, 784 proteins were significantly (*q* < 0.05) differentially abundant in at least in one of the time points when compared between the inoculated- CR and CS root proteomes of parent and progeny across the three time points (Supplementary Table 3). Finally, while there were 684 differentially abundant proteins were common to both CR and CS control samples, 99 proteins demonstrated significant (*q* < 0.05) differential abundance only in the inoculated CR and CS samples, including both progeny and parent lines (Supplementary Table 4). In the pool of unique proteins, there were 36 proteins that showed similar pattern of accumulation in parent and progeny, of them 20 proteins were increased in accumulation and 16 were decreased as the infection progressed to 21 DPI (Supplementary Table 4). In this pool, nine putative proteins were potentially involved in plant and pathogen interaction, proteins with increased accumulation included: orthologs such as fascilin-like (BnaA02T0010200WE), annexin (BnaA06T0293900WE), glutathione S-transferase (Bna A03T0164200WE and BnaA03T0240300WE); and proteins with decreased accumulation pattern included: heat shock protein (Bnascaffold286T0010300WE and BnaA01T01 12500WE), patellin-4-like (BnaC05T0253300WE), ankyrin (BnaA01T0020600WE), and thioredoxin (BnaA03T0276100WE) (Supplementary Table 4).

**TABLE 1:** Sample description and protein run information.

Sample description	Condition	AVG precursors	AVG peptides	AVG protein groups	AVG proteins
Progeny 14 DPI control resistant	PG14CR	8539.00	6351.67	1904.33	3431.67
Progeny 14 DPI control susceptinle	PG14CS	8526.67	6427.67	1902.67	3496.33
Progeny 14 DPI inoculated resistant	PG14IR	7812.33	5768.67	1778.67	3088.67
Progeny 14 DPI inoculated susceptinle	PG14IS	9049.33	6794.33	2010.67	3657.67
Progeny 21 DPI control resistant	PG21CR	7829.67	5871.33	1797.33	3216.67
Progeny 21 DPI control susceptinle	PG21CS	9106.67	6774.00	2024.33	3689.33
Progeny 21 DPI inoculated resistant	PG21IR	7593.33	5670.33	1769.67	3077.67
Progeny 21 DPI inoculated susceptinle	PG21IS	9249.33	6881.33	2011.33	3703.33
Progeny 7 DPI control resistant	PG7CR	9775.00	7162.67	2061.67	3779.00
Progeny 7 DPI control susceptinle	PG7CS	8518.00	6295.00	1908.67	3408.33
Progeny 7 DPI inoculated resistant	PG7IR	8608.67	6462.00	1936.67	3539.33
Progeny 7 DPI inoculated susceptinle	PG7IS	8618.67	6360.67	1881.33	3343.00
Parent 14 DPI control resistant	PR14CR	5336.00	4044.33	1320.67	2296.33
Parent 14 DPI control susceptinle	PR14CS	7720.33	5841.33	1779.00	3110.00
Parent 14 DPI inoculated resistant	PR14IR	7889.67	5828.00	1762.67	3118.33
Parent 14 DPI inoculated susceptinle	PR14IS	7001.67	5223.00	1621.67	2797.00
Parent 21 DPI control resistant	PR21CR	9818.33	7351.67	2121.00	3922.00
Parent 21 DPI control susceptinle	PR21CS	6334.00	4900.00	1533.33	2735.67
Parent 21 DPI inoculated resistant	PR21IR	6497.33	4930.00	1543.67	2788.33
Parent 21 DPI inoculated susceptinle	PR21IS	8413.33	6325.00	1910.00	3474.67
Parent 7 DPI control resistant	PR7CR	6483.67	4927.00	1603.67	2753.33
Parent 7 DPI control susceptinle	PR7CS	7494.33	5672.00	1718.00	3151.67
Parent 7 DPI inoculated resistant	PR7IR	8560.00	6394.67	1923.33	3532.67
Parent 7 DPI inoculated susceptinle	PR7IS	9224.33	6784.67	2001.00	3652.33

### Temporal Changes in the Root Proteome of Clubroot Resistant and Clubroot Susceptible Progeny Lines in Response to the Pathogen

We profiled the proteome level differences that were induced in the CR- and CS-progeny lines in response to the pathogen at the three time points. In this study, comparisons were made between the uninoculated controls and pathogen-challenged CR- and CS-plants at these time points.

#### 7 Days Post Inoculation

**FIGURE 4:**
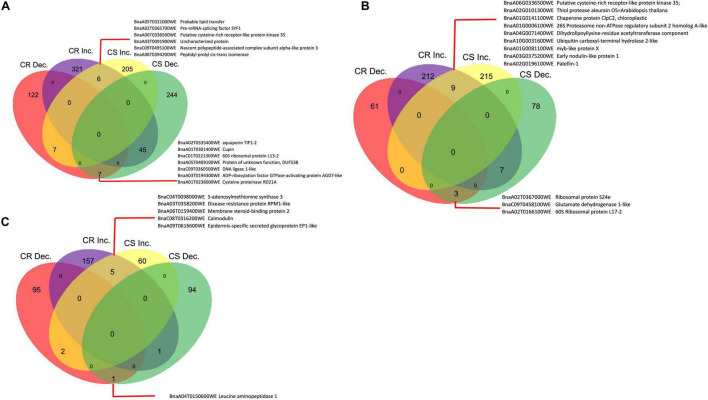
Differentially accumulated proteins (DAP) between control (CC) and inoculated (In), In/CC CR and In/CC CS in the progeny lines. The numeric values indicate the number of proteins that were significantly changing in abundance (*q*-value < 0.05) in protein abundance. **(A)** 7 DPI, **(B)** 14 DPI, and **(C)** 21 DPI. CR, clubroot resistant; CS, clubroot susceptible; Inc and Dec represent Increase and Decrease; respectively.

At 7 DPI, there were 321 and 205 DAPs that increased in abundance in CR- and CS- lines, respectively (Figure 4A); whereas 122 and 244 were decreased in abundance in the CR- and CS- lines, respectively in response to the pathogen (Figure 4A). Among these, six proteins increased, while seven decreased in abundance in both lines (Figure 4A).

Among the proteins that exhibited similar trend of change in abundance in both CR- and CS-progeny lines, hereinafter referred to as ‘shared proteins’ (Supplementary Table 2-4A). Plant and pathogen interaction related proteins such as major intrinsic protein (aquaporin TIP1-2) (BnaA02T0335400WE) and germin-like protein (BnaA01T0301400WE) from the cupin family were observed to decrease in abundance in both CR and CS lines. In contrast, cysteine rich receptor like protein kinase (BnaA06T0336500WE) involved in salt and antifungal stress responses was observed to increase in abundance in both the CR- and CS-progeny lines following pathogen challenge (Supplementary Table 2-4A).

In addition to the “shared proteins” above, we also identified the proteins that exhibited an increase in abundance in the CR- and decreased in the CS-progeny lines and vice-versa, hereinafter referred to as “contrasting proteins.” There were 52 DAPs that exhibited these contrasting trends in abundance in the progeny lines (Supplementary Table 5-4A). These DAPs that increased in the CR- and decreased in CS-progeny lines included stress related proteins, for instance, ferredoxin (BnaC03T0212500WE), peroxidase (BnaC09T0553700WE), chitin elicitor-binding protein (BnaC03T0429600WE), and remorin (BnaC08T0350700WE) (Supplementary Table 5-4A). Other proteins such as thaumatin (BnaC08T0208400WE and BnaA08T0229700WE) and peroxidase C3 (BnaC04T0547800WE) were observed to be decreased in the CR- and increased in the CS-progeny lines in response to the pathogen infection (Supplementary Table 5-4A).

#### 14 Days Post Inoculation

When a similar comparison was made at 14 DPI, there were 212 and 215 DAPs, that increased in abundance in CR- and CS-progeny lines, respectively, whereas 61 and 78 decreased in the CR- and CS-progeny lines, respectively, following pathogen challenge (Figure 4B). Nine proteins increased and three proteins decreased in abundance in both CR- and CS-progeny lines (Figure 4B). Seven proteins demonstrated contrasting trends in the CR- and CS- lines including one protein (BnaA05T0429000WE), orthologous to coiled coil domain which increased in abundance in the CR-progeny lines and decreased in the CS-progeny line (Supplementary Table 5-4B).

In the pool of “shared proteins” between CR- and CS-progeny lines, putative protein, BnaA06G0336500WE (orthologous to cysteine rich receptor like kinase) increased in abundance by >3.93 log_2_ fold in the CR-progeny line in response to the pathogen (Supplementary Table 2-4B). Similarly, two proteins, BnaA10G0031600WE (orthologous to ubiquitin) and BnaA01G0006100WE (orthologous to 26S proteasome) increased significantly in both the CR- and CS-progeny lines (Supplementary Table 2-4B). Other plant stress related proteins orthologous to patellin (BnaA02G0196100WE), chaperone (BnaA01G0141100WE), cathepsin (BnaA02G0101300WE) were also observed to be significantly increased in both CR- and CS-progeny lines in which the relative accumulation of the first two were higher in CR-progeny lines whereas the latter (BnaA02G0101300WE) was higher in CS-progeny lines (Supplementary Table 2-4B).

#### 21 Days Post Inoculation

At 21 DPI, there were 157 and 60 DAPs that increased in abundance in CR- and CS-progeny lines, respectively, whereas 95 and 94 DAPs decreased in abundance in CR- and CS-progeny lines, respectively, following pathogen challenge (Figure 4C). Five proteins were found to increase in both CR- and CS-progeny lines (Supplementary Table 2-4C), while three proteins exhibited contrasting trends in abundance in the CR- and CS-progeny lines including one of the putative proteins (BnaC06T0464800WE) orthologous to metacaspase-4 which was increased in the CR- and decreased in the CS-progeny line (Supplementary Table 5-4C). In the pool of shared proteins, proteins orthologous to calmodulin (BnaC08T0316200WE), disease resistance protein (RPM1-like) (BnaA03T0358200WE), *s*-adenosylmethionine synthase (BnaC04T0098000WE), and epidermis specific secreted glycoprotein (BnaA09T0616600WE) were increased significantly in both CR- and CS-progeny lines (Supplementary Table 2).

### Temporal Changes in the Root Proteome of Clubroot Resistant and Clubroot Susceptible Parent Lines in Response to the Pathogen

In addition to the aforementioned comparison of the pathogen-induced, temporal proteome changes at the three different time points CR- and CS-progeny lines, we also performed a similar study in the CR- and CS-parent lines. Similar to the progeny lines, in this case, comparisons were made between the uninoculated controls and pathogen-challenged CR and CS plants at these time points.

#### 7 Days Post Inoculation

**FIGURE 5:**
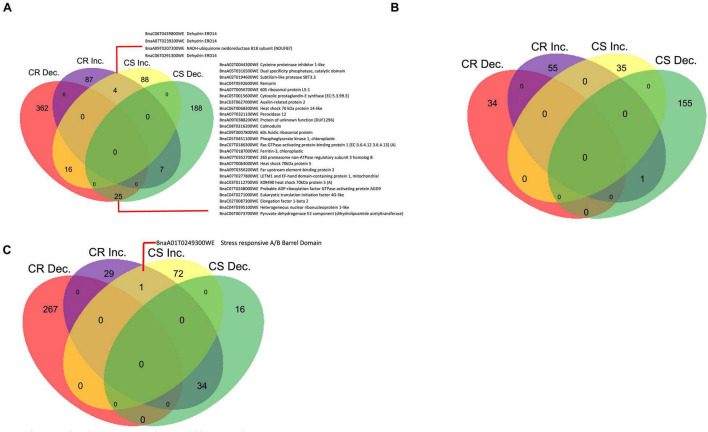
Differentially accumulated proteins (DAP) between control (CC) and inoculated (In), In/CC CR and In/CC CS in the parent lines. The numeric values indicate the number of proteins that were significantly changing in abundance (*q*-value < 0.05) in protein abundance. **(A)** 7 DPI, **(B)** 14 DPI, **(C)** 21 DPI. CR, clubroot resistant; CS, clubroot susceptible; Inc and Dec represent Increase and Decrease; respectively.

At 7 DPI, we observed 87 and 88 proteins that increased in abundance in CR and CS-parent lines, respectively, and 362 and 188 proteins were observed to decrease in CR- and CS-parent lines, respectively, in response to the pathogen (Figure 5A). In addition, we observed that four proteins increased in both CR- and CS-parent lines and 25 proteins decreased in both parent lines (Figure 5A). Among these, proteins orthologous to dehydrin (BnaC06T0291300WE, BnaA07T02 39200WE, and BnaC06T0439800WE) were increased in abundance in the root proteome of the CS-parent line, while cysteine proteinase inhibitor (BnaA02T0044300WE), remorin (BnaC04T0592600WE), auxilin (BnaC03T0627000WE), heat shock 70 kDa (BnaC06T0068300WE and BnaC03T0112700WE), calmodulin (BnaC08T0316200WE), and ferritin (BnaA07T01 87000WE) were decreased in abundance in both the CR- and CS-parent lines in response to the pathogen (Supplementary Table 2-5A).

Among the contrasting proteins, there were 27 DAPs that exhibited opposing trends in abundance following pathogen inoculation at 7 DPI (Supplementary Table 5-5A). They included stress related proteins, for instance, cysteine proteinase RD19a (Bnascaffold1716T0016400WE), calnexin (BnaA06T0265000WE), allene oxide cyclase 4 (BnaA08T0253000WE), peroxidases (BnaC01T0310600WE and BnaA05T0420500WE) (Figure 5A and Supplementary Table 5-5A). The latter two peroxidases and RD19a were increased in the CS lines and decreased in the CR lines; whereas the calnexin and AOC were decreased in the CS line and increased in the CR line.

#### 14 Days Post Inoculation

When a similar comparison was made at 14 DPI, there were 55 and 35 DAPs increased in abundance in CR- and CS- parent lines, respectively, whereas 34 and 155 DAPs decreased in abundance in the CR- and CS- parent lines, respectively in response to the pathogen (Figure 5B). One of the putative proteins (BnaC06T0291300WE) orthologous to dehydrin ERD14 demonstrated a contrasting pattern, increasing in the CR and decreasing in the CS-parent line (Figure 5B and Supplementary Table 5-5B). No shared proteins were detected in this time point.

#### 21 Days Post Inoculation

At 21 DPI, when the root proteomes of CR- and CS-parent lines were compared, we observed 29 and 72 DAPs that were increased in abundance in CR- and CS-parent lines, respectively, whereas 267 and 16 DAPs decreased in abundance in CR- and CS-parent lines, respectively, following pathogen challenge (Figure 5C). One protein (BnaA01T0249300WE), orthologous to stress responsive A/B barrel domain, was observed to increase in both CR- and CS-parent lines (Supplementary Table 2-5C), whereas 34 proteins exhibited contrasting trends in abundance in the CR- and CS-parent lines including ten putative proteins that are potentially involved in mediating plant and pathogen responses (Figure 5C and Supplementary Table 5-5C). These contrasting proteins included fasciclin-like arabinogalactan (BnaA01T0140800WE, BnaA10T0023700WE, and BnaA09T0184900WE), KDEL-tailed cysteine endopeptidase (BnaA06T0163700WE), thioredoxin-like domain (BnaA09T0477700WE), peroxidase (BnaA09T0646900WE), and patellin-4 (BnaC07T0112600WE) (Supplementary Table 5-5C). All the orthologs were increased in the CR-parent and decreased in the CS-parent lines following pathogen challenge (Figure 5C and Supplementary Table 5-5C).

### Pathogen-Induced Temporal Changes in Root Proteome—A Comparison of Clubroot Resistant/Clubroot Susceptible Progeny and Clubroot Resistant/Clubroot Susceptible Parent Lines

In this case, we compared the pathogen-induced proteome level changes in the CR-parent and CR-progeny lines against their respective CS-counterparts. In other words, we compared the pathogen-induced temporal changes in the proteome of the CR-progeny against similar changes taking place in the CS-progeny lines and the CR-parent against the CS-parent lines at the three different time points. Our results at each time point are described below.

#### 7 Days Post Inoculation

**FIGURE 6:**
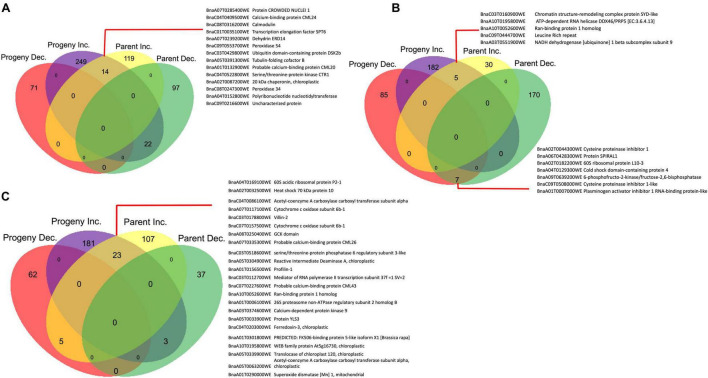
Differentially accumulated proteins (DAP) between clubroot resistant (CR) and susceptible (CS) inoculated, CR/CS progeny and CR/CS parent lines in the progeny and parental lines. The numeric values indicate the number of proteins that were significantly changing in abundance (*q*-value < 0.05) in protein abundance. **(A)** 7 DPI, **(B)** 14 DPI, **(C)** 21 DPI. Inc and Dec represent Increase and Decrease; respectively.

At 7 DPI, 249 and 119 DAPs increased in abundance in the CS-progeny and CS-parent lines, respectively following pathogen challenge (Figure 6A). While 71 and 97 DAPs decreased in abundance in the CS-progeny and CS-parent lines, respectively (Figure 6A). There were 14 shared proteins increased in both CR-progeny and in the CR-parent, which included stress responsive metabolic compounds such as, calmodulin (BnaC08T0316200WE), dehydrin ERD14 (BnaA07T0239 200WE), peroxidase (BnaC09T0553700WE, BnaC08T0247 300WE), and calcium binding protein (BnaC04T0409500WE) (Figure 6A and Supplementary Table 3-6A).

#### 14 Days Post Inoculation

When a similar comparison was made at 14 DPI, 182 and 30 proteins increased in abundance in the CS-progeny and CS-parent lines, respectively, following pathogen challenge (Figure 6B). While 85 and 170 proteins decreased in abundance in the CS-progeny and CS-parent lines, respectively (Figure 6B). There were five shared proteins that increased in abundance in both the CS-progeny and the CS-parent, including a protein orthologous to leucine rich repeat domain (BnaC09T0444700WE), RNA binding protein 1 (BnaA10T0052600WE), and chromatin structure-remodeling complex protein SYD-like (BnaC03T0160900WE) (Supplementary Table 3-6B). One of the putative proteins (BnaA03T0551900WE) orthologous to NADH dehydrogenase [ubiquinone] 1 beta subcomplex subunit 9 increased > 9.0 log_2_ fold in both CR-progeny and CR-parent lines (Supplementary Table 3-6B). Among the shared proteins, there were seven proteins increased in abundance in both CS-progeny and CS-parent group. These proteins included the orthologs of cysteine proteinase inhibitor (BnaA02T00443 00WE and BnaC09T0508000WE), SPIRAL1-like protein (BnaA06T0428300WE), Cold shock domain-containing protein (BnaA04T0129300WE) and 60S ribosomal protein (BnaA02T0182200WE) (Supplementary Table 3-6B).

#### 21 Days Post Inoculation

At 21 DPI, there were 181 and 107 DAPs that were increased in the CR-progeny and CR-parent lines, respectively; while 62 and 37 were decreased in the CR-progeny and CR-parent lines, respectively (Figure 6C). Twenty-three proteins were commonly increased in CR-progeny and CR-parent lines, nine of them were orthologous to stress response proteins including heat shock 70 (BnaA02T0032500WE), villin (BnaC03T0178800WE), calcium binding protein (CML43) (BnaC07T0227600WE), calcium dependent kinases (BnaA03T0374600WE), ferredoxin (BnaC04T0203000WE), and superoxide dismutase (Figure 6C). One of the proteins orthologous to superoxide dismutase [Mn] 1 (BnaA01T0290000WE) was increased > 2 folds in both CR-progeny and CS-parent lines (Supplementary Table 3-6C).

### Gene Ontology Enrichment Analysis of Differentially Abundant Proteins in the Root Proteomes of Progeny Lines

The DAPs that were identified in the various comparisons described above, that were temporally regulated as a result of pathogen challenge in the CS-progeny, CR-progeny, CS-parent, and CR-parent were further analyzed for their potential biological functions. The results obtained with respect to the enriched categories in the GO biological processes and molecular functions are provided in Supplementary Tables 6A–D. Our key findings are described below.

### Clubroot Resistant-Progeny

By 21 DPI, 58 unique GO terms associated with various biological processes were significantly (FDR < 0.05) enriched in the CR-progeny lines (Supplementary Table 6A). The vast majority of these biological processes included: cellular process (GO:0009987), response to stimulus (GO:0050896), protein metabolic process (GO:0019538), and response to stress (GO:0006950) in the CR-progeny lines (Supplementary Table 6A). In addition, there were several categories that are associated with plant-microbe interactions including: response to - biotic stimulus (GO:0009607), – bacterium (GO:0009617), – other organism (GO:0051707), -oxidative stress (GO:0006979), and – ROS (GO:0000302) (Supplementary Table 6A).

In terms of molecular functions, there were 24 enriched categories in the CR-progeny lines (Supplementary Table 6A). Categories that were highly enriched included: ion binding (GO:0043167), cation binding (GO:0043169), and calcium ion binding (GO:0005509) (Supplementary Table 6A). Molecular function categories related to ROS such as oxidoreductase (GO:0016491), antioxidant activity (GO:0016209), acting on peroxide as acceptor (GO:0016684), and peroxidase activity (GO:0004601) were greatly enriched indicating their potential involvement in signal transduction processes in response to the pathogen in the CR-progeny lines.

### Clubroot Susceptible-Progeny

By 21 DPI, 59 unique GO terms associated with biological processes were significantly (FDR < 0.05) enriched in the CS-progeny lines (Supplementary Table 6B). The highest-level categories included response to -stimulus (GO:0050896), -stress (GO:0006950), and -abiotic stimulus (GO:0009628) (Supplementary Table 6B). Similar to the results described above for the CR-progeny lines, there were several categories directly associated with plant-microbe interactions such as response to -other organism (GO:0051707), defense response (GO:0006952), and – biotic stimulus (GO:0009607) (Supplementary Table 6B).

In terms of molecular functions, there were 18 enriched categories in the CS-progeny lines (Supplementary Table 6B). Categories that were enriched included: ion binding (GO:0043167), cation binding (GO:0043169), metal ion binding (GO:0046872), oxidoreductase activity (GO:0016491), and structural molecule activity (GO:0005198) (Supplementary Table 6B). Molecular function categories related to ROS, for instance, antioxidant activity (GO:0016209), oxidoreductase (GO:0016491), acting on peroxide as acceptor (GO:0016684), and intramolecular oxidoreductase activity (GO:0016860) were highly enriched indicating their potential role in plant and pathogen interaction in the CS-progeny lines as well (Supplementary Table 6B). In addition, GO categories related to metal binding such as calcium ion- (GO:0005509), copper ion- (GO:0005507), heme- (GO:0020037), and iron ion binding (GO:0005506) were highly enriched (Supplementary Table 6B).

### Shared and Contrasting Gene Ontology Categories in the Clubroot Resistant and Clubroot Susceptible Progeny Lines

In terms of biological process, 39 categories in the CR- and CS-progeny lines were similar and majority of them are potentially associated with plant-pathogen interactions, including GO categories in response to – stress (GO:0006950), – biotic stimulus (GO:0009607), – bacterium (GO:0009617), – other organism (GO:0051707), – ROS (GO:0000302), and defense response (GO:0006952).

Nineteen GO categories were unique to the root proteome of the CR-progeny lines including cellular process (GO:0009987), which was one of the highest enriched categories in the CR progeny (Supplementary Tables 6A,B). Other categories such as protein metabolic process (GO:0019538), cellular protein metabolic process (GO:0044267), translation (GO:0006412) were also among the unique categories in the CR-progeny lines (Supplementary Tables 6A,B). Eleven GO categories were unique to CS-progeny including defense response (GO:0006952), cellular component biogenesis (GO:0044085), and cellular component assembly (GO:0022607) (Supplementary Tables 6A,B).

In terms of molecular functions, 17 GO categories were identical in the CS and CR progeny including ion binding (GO:0043167), metal ion binding (GO:0046872), and structural molecule activity (GO:0005198) (Supplementary Tables 6A,B). The GO categories such as oxidoreductase activity (GO:0016491) and acting on sulfur group of donors (GO:0016667) were the only GO terms unique to the CS-progeny line (Supplementary Table 6B). Similarly, seven unique GO categories were found in CR-progeny line (Supplementary Table 6A) including binding (GO:0005488), endopeptidase activity (GO:0004175), translation factor activity, and nucleic acid binding (GO:0008135) (Supplementary Table 6A).

### Gene Ontology Enrichment Analysis of Differentially Abundant Proteins in the Root Proteomes of Parent Lines

#### Clubroot Resistant-Parent

By 21 DPI, 55 unique GO terms associated with biological processes were significantly (FDR < 0.05) enriched in the root proteome of the CR-parent line (Supplementary Table 6C). The highest-level categories included cellular process (GO:0009987), response to -stimulus (GO:0050896), -stress (GO:0006950), cellular protein metabolic process (GO:0019538), and response to chemical stimulus (GO:0042221) in the parent lines (Supplementary Table 6C). There were several categories associated with plant-microbe interactions including response to - biotic stimulus (GO:0009607), -bacterium (GO:0009617), -other organism (GO:0051707), and response to ROS (GO:0000302) that were observed. Similarly, enriched categories associated with response to -metal ion (GO:0010038), and -cadmium ion (GO:0046686) were also observed to be highly enriched (Supplementary Table 6C).

In terms of molecular functions, there were 22 enriched categories in the root proteome of the CR-parent line in response to the pathogen (Supplementary Table 6C). These highly enriched categories include binding (GO:0005488), ion binding (GO:0043167), cation binding (GO:0043169), and metal ion binding (GO:0046872) (Supplementary Table 6B). Molecular function categories related to ROS such as antioxidant activity (GO:0016209), peroxidase activity (GO:0004601), and intramolecular oxidoreductase activity (GO:0016860) were highly enriched. Similarly, GO categories associated with calcium ion binding (GO:0005509) and heme binding (GO:0020037) were among some of the highest-level enriched categories in the molecular function (Supplementary Table 6C).

#### Clubroot Susceptible-Parent

By 21 DPI, 36 unique GO terms associated with biological processes were significantly (FDR < 0.05) enriched in the CS-parent line (Supplementary Table 6D). GO enriched categories in the root proteome of the CS-parent line was similar to that of the CR-parent line. The highest-level categories included response to -stimulus (GO:0050896), -stress (GO:0006950), and protein metabolic process (GO:0019538) (Supplementary Table 6D). There were several categories directly associated with plant-microbe interactions such as response to – other organism (GO:0051707), – defense response (GO:0006952), -biotic stimulus (GO:0009607), and -bacterium (GO:0009617) (Supplementary Table 6D).

In terms of molecular function, there were 22 enriched categories in the CS-parent lines. Categories including binding (GO:0005488), cation binding (GO:0043169), ion binding (GO:0043167), and metal ion binding (GO:0046872) were highly enriched in the CS-parent line in response to the pathogen challenge (Supplementary Table 6D). Molecular function categories related to ROS such as antioxidant activity (GO:0016209), peroxidase activity (GO:0004601), and oxidoreductase (GO:0016491) were highly enriched (Supplementary Table 6D). Likewise, metal ion binding related categories, such as calcium ion binding (GO:0005509) and heme binding (GO:0020037) were also highly enriched (Supplementary Table 6D).

### Shared and Contrasting Gene Ontology Categories in the Clubroot Resistant and Clubroot Susceptible Parent Lines

In terms of biological process, 34 categories in the root proteome of the CR-parent and the CS-parent line were identical and majority of them were potentially associated with plant and pathogen interaction, including GO categories in response to -stimulus (GO:0050896), -stress (GO:0006950), cellular protein metabolic process (GO:0019538), and response to chemical stimulus (GO:0042221) (Supplementary Tables 6C,D). There were also several categories associated with plant-microbe interactions such as response to – biotic stimulus (GO:0009607), -bacterium (GO:0009617), and -oxidative stress (GO:0006979) that were observed (Supplementary Tables 6C,D).

Three GO categories were unique to the root proteome of the CS-parent line including protein metabolic process (GO:0019538), protein import (GO:0017038), and protein localization in organelle (GO:0033365) (Supplementary Tables 6C,D). Similarly, 22 GO categories were unique to CR-parent including cellular process (GO:0009987), post-embryonic development (GO:0009791), and response to metal ion (GO:0010038) (Supplementary Tables 6C,D).

In terms of molecular function, 22 GO categories were identical in the root proteome of the CS- and CR-parent line including binding (GO:0005488), ion binding (GO:0043167), cation binding (GO:0043169), and metal ion binding (GO:0046872) (Supplementary Tables 6C,D). The GO terms, oxidoreductase activity (GO:0016491) and isomerase activity (GO:0016853) were unique in the root proteome of the CR-parent line (Supplementary Table 6C). Similarly, two unique GO categories, *s*-acyltransferase activity binding (GO:0016417) and cysteine-type endopeptidase activity were found in the root proteome of the CS-parent line (Supplementary Table 6D).

In the following sections, we describe the major groups of proteins that were found to be differentially abundant, and which are potentially involved in mediating the resistance phenotype in the CR lines used in this study.

### Plant Hormone Related Proteins

Due to the important roles of phytohormones in the progression of clubroot disease in *B. napus*, we analyzed our proteome data for DAPs associated with phytohormone biosynthesis and/or signaling. When the control and inoculated lines were compared, i.e., inoculated vs. control-CR and inoculated vs. control -CS lines, in the case of both progeny and parents, there were six DAPs that were related to salicylic acid (SA) regulation. Four of them (BnaA03T0555800WE, BnaC07T0044900WE, Bnascaffold286T0028200WE, and BnaA01T0310200WE) were orthologous to *Arabidopsis* regulatory protein, non-expressor of pathogenesis related 1 (NPR1) and NPR4, whereas two of them (BnaA09T0109100WE and BnaA10T0130500WE) were orthologous to systematic acquired resistance (SAR) deficient 1 protein (Supplementary Table 7A). Three of the proteins [(Bnascaffold286T0028200WE and BnaC07T004 4900WE, orthologous to regulatory protein NPR1), and (BnaA02T0079400WE, orthologous to aminotransferase ALD1)] showed consistent pattern in both parent and progeny lines. Two of the NPR1 orthologs (Bnascaffold286T0028200WE BnaC07T0044900WE) showed contrasting pattern between CR and CS lines, thereby the proteins increase in CR and decrease in CS in both the parent and progeny lines, while the protein BnaA02T0079400WE consistently increased in accumulation in both CR and CS root proteomes across both parent and progeny lines (Supplementary Table 7A).

There were 12 DAPs that were orthologous to proteins related to ethylene (ET) and jasmonic acid (JA) biosynthetic pathways (Supplementary Table 7A). Seven DAPs were orthologous to serine/threonine-protein kinase CTR1, which functions as a negative regulator of the ET biosynthetic pathway (Kieber et al., 1993). The protein, (BnaA06T0336500WE) orthologous to serine/threonine-protein kinase CTR1, increased in abundance in both CR and CS and the pattern was consistent in both the parent and progeny lines (Supplementary Table 7A). Similarly, there were three proteins (BnaA09T0013600WE, BnaA03T0361400WE, and BnaC04T0098000WE) orthologous to *s*-adenosylmethionine (SAM) synthase that exhibited a similar pattern of protein accumulation, i.e., an increase in both CR and CS, in both the parent and progeny lines (Supplementary Table 7A). As the pathogen infection progressed to 21 DPI, the protein, BnaA03T0361400WE, increased in the CR lines and decreased in abundance in the CS lines in both the parent and progeny (Supplementary Table 7A). Similarly, the proteins, BnaA09T0013600WE and BnaC04T0098000WE, were increased in abundance in the CR lines in both the parent and progeny and the increasing pattern was similar in CS-progeny, however, the pattern in CS parent at 21 DPI was decreasing (Supplementary Table 7A).

Two plant hormones, auxin and cytokinins (CK), are involved in clubroot gall formation. We identified three auxin related proteins (BnaA10T0208100WE, BnaA05T040 9700WE, and BnaC03T0302500WE) and three CK related proteins (BnaC07T0399800WE, BnaA06T0322500WE, and BnaA01T0059700WE) (Supplementary Table 7A). As the infection progressed to 21 DPI, one of the putative proteins (BnaA05T0409700WE) orthologous to auxin-induced in root cultures protein 12 was increased in abundance in both CR and CS root proteomes and the pattern was similar in both the parent and progeny lines (Supplementary Table 7A). Similarly, one of the orthologs of histidine kinase (BnaA01T0059700WE) also increased at 21 DPI, showing shared pattern between CR-progeny, CR-parent, and CS-progeny; however, in the CS-parent, the protein decreased in abundance at 21 DPI (Supplementary Table 7A).

In an addition to the comparisons mentioned above, root proteomes were compared between inoculated CR-parent vs. inoculated CS-parent and inoculated CR-progeny vs. inoculated vs. CS-progeny. There were eight proteins representing five important phytohormones, including auxin, CK, ET, JA, and SA (Supplementary Table 7B). Two SA related proteins (BnaC07T0044900WE and Bnascaffold286T0028200WE) were detected in both CR and CS root proteomes, and the latter showed a shared pattern with an increase in both the CR-parent and CR-progeny as the infection progressed to 21 DPI (Supplementary Table 7B). Other proteins involved in ET mediated (BnaA09T0013600WE) and JA mediated (BnaA08T0253000WE) defense responses were increased in the CR-progeny at all three times points. Four proteins related to phytohormones, auxin (BnaA05T0409700WE, BnaC03T03 02500WE) and CK (BnaC07T0399800WE, BnaA01T0059 700WE) were differentially abundant in both parent and progeny lines (Supplementary Table 7B). While both the CK related proteins were increased in CR-progeny, one CK related protein (BnaA01T0059700WE), that is orthologous to a histidine kinase, was consistently increased in both the CR- parent and progeny lines at 21 DPI (Supplementary Table 7B). Similarly, the auxin related protein (BnaA05G0409700WE), orthologous to auxin-induced in root cultures protein 12, also exhibited a shared pattern of abundance and increased in both CR-parent and CR-progeny lines (Supplementary Table 7B).

### Proteins Involved in Calcium Signaling

When the root proteomes were compared between the inoculated vs. control CR and inoculated vs. control CS lines, 32 differentially accumulated proteins orthologous to calcium signaling proteins were detected (Supplementary Table 8A). Among these proteins, 21 contained helix-loop-helix structural motifs and were categorized as EF-hand domain containing proteins, nine were labeled as calreticulin family, and the remaining two were related to calmodulin binding protein (Supplementary Table 8A). As the infection progressed to 21 DPI, three putative calcium signaling proteins (BnaA07T0335300WE, BnaA03T0464700WE, and BnaA03T0374600WE) were consistently increased in the root proteome of the CR-progeny and CR-parent lines (Supplementary Table 8A). In contrast, all three proteins consistently decreased in both the CS-parent and CS-progeny lines at 21 DPI (Supplementary Table 8A).

In an addition to the comparison mentioned above, DAPs were assessed between inoculated CR-parent vs. inoculated CS-parent and inoculated CR-progeny vs. inoculated CS-progeny and 22 orthologs of calcium signaling proteins were identified (Supplementary Table 8B). Three of these putative proteins (BnaC08T0316200WE, BnaC07T0227600WE, and BnaA07T0335300WE), were orthologous to calcium binding proteins, and were consistently increased at all three time points in both the CR-parent and CR-progeny lines (Supplementary Table 8B).

### Lignin Related Proteins

**FIGURE 7:**
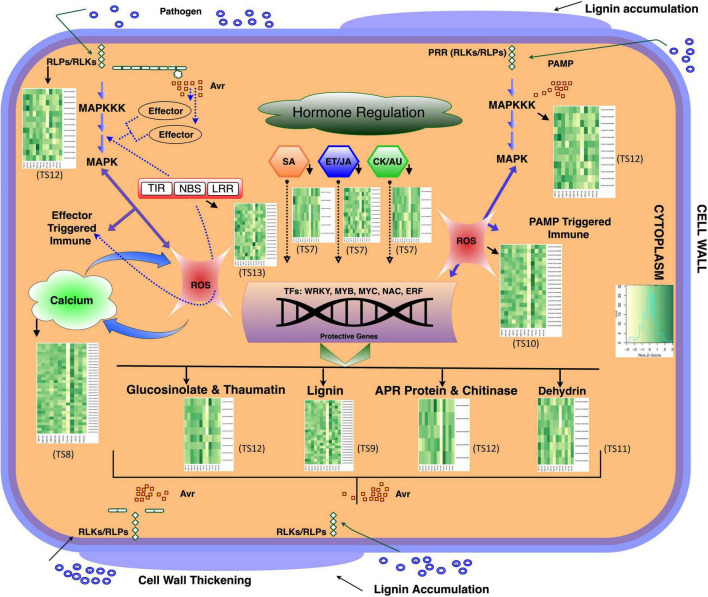
Schematic diagram of plant-pathogen interaction upon *P. brassicae* inoculation in *B. napus*. Heatmaps were developed using log_2_ fold change values at 7-, 14-, and 21-DPI and were shown besides each major metabolic processes in the plant-pathogen interaction pathway indicated by a dark solid arrow. Dark Green indicates increase in abundance and light color indicates decrease in abundance. Predicted proteins that were included in the heatmaps were significantly (*q* < 0.05) differentially abundant at least in one time point across all genotypes investigated. During *P. brassicae* infection, pathogen releases signal metabolites such as PAMPs and effectors and these elicitors were recognized by host cells. Plant receptors such as receptor like -kinases and -proteins (RLKs/RLPs) and resistance (R) proteins interact with the pathogen signal compounds, thereby activates the MAP kinases (signal transduction). Transcription factors are regulated and successively plant defense related metabolic pathways are activated, proteins related to phytohormone biosynthesis pathway, especially SA, JA, and ET biosynthetic pathway and other secondary metabolism were activated. Pathogen defense related secondary metabolites such as glucosinolates, small molecules thaumatin, polyssacharides (lignin), glycosyl hydrolases (chitinases), and a spectrum of antimicrobial and defense related proteins including dehydrin proteins were differentially accumulated in response to the pathogen. Growth regulators, auxin (AU) and cytokinin (CK) were manipulated by the pathogen, the hormones are regulated abnormally, thereby leading to the hypertrophy in the root tissues. There is an interplay between ROS and calcium signaling compounds during plant and pathogen (*P. brassicae*) interaction. Calcium acts as a second messenger and with the involvement of Ca^2+^ -pumps, -buffers, and –exchangers, they orchestrates and regulates cellular processes and participates in an interplay with other signaling transduction processes such as ROS. Detail annotation of protein ids shown in the heatmaps are provided in the supplementary tables: TS7 – TS13 (Supplementary Tables 7–13). Avr, avirulence factor; RLP, receptor like proteins; RLK, receptor like kinase; SA, salicylic acid; JA, jasmonic acid; ET, ethylene; CY, cytokinin; AU, auxin; PRR, pathogen recognition receptor; PAMP, pathogen associated molecular pattern; MAPK, mitogen-activated protein kinase; TIR, toll/interleukin-1 receptor; NBS, nucleotide binding site; LRR, leucine rich repeat; APR, adult plant resistance. PgR-7d, CR progeny 7 DPI; PgR-14d, CR progeny 14 DPI; PgR-21d, CR progeny 21 DPI; PgS, CS progeny; PrR, CR parent; PrS, CS parent.

The comparative analysis of the root proteomes between the inoculated vs. control CR and inoculated vs. control CS lines revealed that there were 24 DAPs that were orthologous to peroxidases, caffeic acid 3-*O*-methyltransferase, lignin forming anionic peroxidase, and *s*-adenosylmethionine (SAM) synthase 3 (METK3) (Figure 7 and Supplementary Table 9A). The enzyme METK3 is involved in lignin biosynthesis (Shen et al., 2002) and there were four protein orthologs for METK3 (BnaC03T0264500WE, BnaA09T0013600WE, BnaA03T0361400WE, and BnaC04T0098000WE), among which, BnaC03T0264500WE, was increased and the pattern was consistent at 21 DPI in CS-progeny and the CS-parent (Supplementary Table 9A). Similarly, there were 13 proteins orthologous to peroxidases, proteins involved in pathogen response as well as during the biosynthesis and degradation of lignin. Among these proteins, three putative proteins (BnaA10T0260700WE, BnaC01T0221000WE, and BnaC08T0247300WE), were increased > 1.0− log_2_ fold in the CR-progeny lines and decreased in the CS-progeny lines when the pathogen infection progressed to 21 DPI (Figure 7 and Supplementary Table 9A). The protein BnaC08T0247300WE (orthologous to peroxidase) was increased in the CS- and CR-parent line, thus exhibiting a trend that was similar to the CR-progeny lines (Supplementary Table 9A).

Root proteomes when compared between inoculated CR-parent vs. inoculated CS-parent and inoculated CR-progeny vs. inoculated CS-progeny, there were 18 DAPs that are related to lignin (Supplementary Table 9B). While majority of the proteins showed contrasting pattern between the CR- parent and progeny lines, three of the proteins (BnaA03T0561300WE, BnaC02T0522200WE, and BnaC08T0247300WE) that are orthologous to peroxidases have shared pattern between parent and progeny, with an increase in the CR root proteomes of both the parents and the progeny lines (Supplementary Table 9B).

### Reactive Oxygen Species Related Proteins

A comparison of the root proteomes between inoculated vs. control CR and inoculated vs. control CS lines indicated that there were 20 putative, differentially abundant, ROS related proteins (Supplementary Table 10A). Five exhibited contrasting trends in abundance between the CR- and CS-progeny lines. Three of these proteins (BnaA09T0646900WE, BnaC04T0547800WE, and BnaC09T0113900WE), orthologous to l-ascorbate peroxidase 5, were decreased in abundance in the CR-progeny and increased in abundance in the CS-progeny lines (Figure 7 and Table S10). The remaining two proteins, BnaA05T0420500WE (orthologous to peroxiredoxin-2F) and BnaA10T0260700WE (orthologous to l-ascorbate peroxidase 5) increased in abundance in the CR-progeny and decreased in the CS-progeny (Figure 7 and Supplementary Table 10). In the case of these two proteins, the pattern of accumulation observed in the CR-progeny lines was also consistent in the CR-parent line with an increase in accumulation as the infection progressed to 21 DPI (Supplementary Table 10A).

Three other ROS-related proteins, BnaA03T0167800WE (orthologous to glutathione peroxidase 2), BnaA06T0044600WE (orthologous to L-ascorbate peroxidase 1), and BnaA09T0631100WE (orthologous to superoxide dismutase) exhibited a similar pattern of protein accumulation in CR-, CS-progeny, and CR-parent lines where the proteins increased in abundance (Supplementary Table 10A). However, two of these proteins (BnaA03T0167800WE and BnaA06T0044600WE) in the CS-parent decreased in abundance as the infection progressed to 21 DPI (Supplementary Table 10A). Nevertheless, among these three proteins, the protein BnaA09T0631100WE (orthologous to superoxide dismutase) has shared pattern of protein abundance between parent and progeny in both the CS and CR lines (Supplementary Table 10A).

When the inoculated CR-parent vs. CS-parent and inoculated CR-progeny vs. inoculated CS-progeny were compared, 31 DAPs were identified. Among them, six proteins [BnaC02T0522200WE-, BnaA03T0561300WE-, and BnaC08T0247300WE-, orthologous to peroxidase; BnaC06T0450900WE, orthologous to thioredoxin-like domain; BnaA01T0290000WE, orthologous to superoxide dismutase; and BnaC07T0044900WE, orthologous to ankyrin repeat domain) exhibited shared patterns between both the parent and progeny lines, with all of them increased in accumulation in the CR lines of both the parents and progeny as the disease progressed to 21 DPI (Supplementary Table 10B). The protein BnaA01T0290000WE increased > 2.0-log_2_ fold change in both CR- parent and CR-progeny lines (Supplementary Table 10B).

### Dehydrins

Between the inoculated vs. control CR and inoculated vs. control CS lines, there were nine proteins orthologous to dehydrin, three of them (BnaC02T0286500WE, BnaC06T0291300WE, and BnaA07T0239200WE) were increased in abundance in the CR-progeny and decreased in the CS-progeny lines (Supplementary Table 11A). All three proteins increased in abundance at 7 DPI in the CS-progeny lines; however, as the infection progressed, they were consistently decreased at 14 and 21 DPI, demonstrating an opposite pattern of protein accumulation relative to the CR-progeny lines (Supplementary Table 11A). In the case of BnaA07T0239200WE, we observed that it was increased in abundance in both the CR-progeny as well as the CR-parent lines as the infection progressed to 21 DPI (Supplementary Table 11A).

In a separate comparison of root proteomes between inoculated CR-parent vs. inoculated CS-parent and inoculated CR-progeny vs. inoculated CS-progeny, four orthologs of dehydrin (BnaC06T0291300WE, BnaC06T0439800WE, BnaA 07T0354800WE, and BnaA07T0239200WE) were identified (Supplementary Table 11B). These proteins decreased in the CR root proteomes of both parent and progeny lines as the infection progressed to 21 DPI (Supplementary Table 11B).

### Heat Shock Proteins

A comparison of the root proteomes between inoculated vs. control CR and inoculated vs. control CS lines revealed that there were 16 differentially accumulated proteins that were related to heat shock proteins (Supplementary Table 12A). Three of the proteins (BnaC06T0068300WE, BnaC04T0303200WE, and BnaC06T0195100WE) orthologous to HSP70 and HSP90, were increased as the infection progressed to 21 DPI in CR lines and decreased at 21 DPI in the CS lines in the case of both the parent and progeny lines except for BnaC06T0195100WE, which increased at 21 DPI in CS-parent lines (Supplementary Table 12A).

Furthermore, root proteomes when compared between inoculated CR-parent vs. inoculated CS-parent and inoculated CR-progeny vs. inoculated CS-progeny, we identified 14 heat shock proteins orthologous to HSP70 and HSP90 kDa (Supplementary Table 12B). Four of the proteins (BnaA02T0032500WE, BnaC06T0068300WE, BnaC04T0303200WE, and Bnascaffold286T0010300WE) were consistently increased in the CR-parent and CR-progeny as the infection progressed to 21 DPI Table S12-B).

### Resistance Proteins

When root proteomes were compared between inoculated vs. control CR and inoculated vs. control CS lines, 24 differentially accumulated R proteins were identified. Seven of these proteins (BnaA01T0054600WE, BnaA03T0182000WE, BnaA03T0374600WE, BnaA06T0336500WE, BnaA10T0238 900WE, BnaC04T0522800WE, and BnaC02T0029600WE) were orthologous to the disease resistance X-TIR-NB-LRR-X family and three (BnaC08T0367400WE, BnaC09T0444700WE, and BnaA06T0288700WE) were orthologous to proteins containing leucine rich repeat (LRR) domains and the remaining were orthologous to stress response and calcium dependent protein kinases (Figure 7 and Supplementary Table 13A). Three of these proteins (BnaA01T0054600WE, BnaA03T0374600WE, and BnaA06T0336500WE) orthologous to WRKY19 increased in abundance in the CR-progeny as well as in the CR-parent lines; whereas, the latter two (BnaA01T0054600WE and BnaA06T0336500WE) were consistently increased at all time points in both the CR and CS genotypes (progeny and parent), while one of the proteins (BnaA03T0374600WE) decreased in the CS-parent and CS-progeny as the infection progressed to 21 DPI (Supplementary Table 13A).

When the inoculated CR-parent vs. inoculated CS-parent and inoculated CR-progeny vs. inoculated CS-progeny were compared, 15 DAPs that were related to disease resistance were identified (Supplementary Table 13B). Three proteins orthologous to WRKY19 showed shared pattern between parent and progeny lines; while two of them (BnaA03T0374600WE and BnaC01T0013800WE) were increased in the CR-parent and CR-progeny lines, and BnaA03T0397600WE was decreased in both CR parent and CR progeny as the infection progressed to 21 DPI (Supplementary Table 13B).

### Differentially Abundant Proteins Encoded by Genes Located on Chromosome A3 and A8

Resistance to clubroot has been frequently mapped to QTLs located on chromosomes A3 and A8 (Hasan et al., 2021). When we compared the root proteomes between inoculated vs. control CR and inoculated vs. control CS in both parent and progeny, we detected 30 and 25 DAPs that were located on chromosome A3 and A8, respectively (Supplementary Tables 14A,C).

**FIGURE 8:**
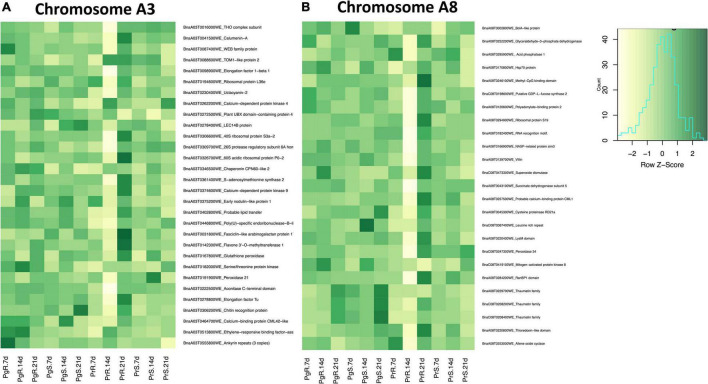
Differentially accumulated proteins (DAP) at all three time points between control (CC) and inoculated (In) lines, In/CC CS and In/CC CR lines across parent and progeny, these proteins are from **(A)** chromosome A3 and **(B)** A8. Heatmaps were developed using log_2_ fold change values at 7-, 14-, and 21-DPI. Dark Green indicates increase in abundance and light color indicates decrease in abundance. Proteins included in the heatmaps were significantly (*q*-value < 0.05) differentially abundant at least in one time point across all genotypes investigated.

Among the DAPs, located on chromosome A3, we identified 15 that were orthologous to a range of plant-pathogen interaction related proteins, including fasciclin-like arabinogalactan (BnaA03T0031800WE), peroxidase (BnaA03T0191900WE), calumenin (BnaA03T0041500WE), chitin recognition protein (BnaA03T0306200WE), and ankyrin (BnaA03T0555800WE) (Figure 8A and Supplementary Table 14A). Five of these proteins increased in abundance by 21 DPI in the CR-progeny lines, with two of the five DAPs, BnaA03T0142300WE (orthologous to flavone 3′-*O*-methyltransferase 1) and BnaA03T0374600WE (orthologous to calcium-dependent protein kinase 9) increased in CR and decreased in CS lines in both the progeny as well as parent lines (Supplementary Table 14A). Similarly, three proteins, BnaA03T0167800WE (orthologous to glutathione peroxidase), BnaA03T0306200WE (chitin recognition protein), and BnaA03T0262200WE (calcium-dependent protein kinase 4) were observed to be increased in both the CR-progeny as well as the CR-parent lines (Supplementary Table 14A).

In separate root proteome comparisons between inoculated CR-parent vs. inoculated CS-parent and inoculated CR-progeny vs. inoculated CS-progeny, there were 26 DAPs that are located on chromosome A3. Four proteins among these, BnaA03T0003400WE (orthologous to mitochondrial ATPase inhibitor), BnaA03T0041500WE (calumenin-A), BnaA03T0402800WE (lipid transfer), and BnaA03T0135900WE (C2 domain) showed consistent pattern of accumulation in parent and progeny lines (Supplementary Table 14B). Three of these proteins (BnaA03T0003400WE, BnaA03T0402800WE, and BnaA03T0041500WE) were increased in the CR-parent and CR-progeny line, and one of the proteins (BnaA03T0135900WE) was decreased in the CR-lines in both parents and the progeny (Supplementary Table 14B).

When root proteomes were compared between inoculated vs. control CR and inoculated vs. control CS in both parent and progeny, 16 putative DAPs were related to plant and pathogen interactions and were located on chromosome A8 (Supplementary Table 14C). Some of the major plant–microbe interaction related proteins included peroxidase (BnaC08T0247300WE), thaumatin (BnaC08T0208400WE, BnaC08T0208200WE, and BnaA08T0229700WE), and villin (BnaA08T0139700WE) (Figure 8B and Supplementary Table 14C). As the infection progressed to 21 DPI, three proteins (BnaA08T0229700WE, BnaC08T0208200WE, and BnaC08T0208400WE) orthologous to thaumatin family proteins were increased by > 2.0-log_2_ fold in the CR progeny lines (Supplementary Table 14C). The pattern of accumulation for the latter two were consistent in the CR parent line as well (Supplementary Table 14C). On the contrary, protein (BnaC08T0247300WE) orthologous to peroxidase 34 exhibited a contrasting pattern, wherein it increased in the CR-progeny and decreased in the CS-progeny lines (Supplementary Table 14C).

Furthermore, when the root proteomes of the inoculated CR-parent vs. inoculated CS-parent and inoculated CR-progeny vs. inoculated CS-progeny were analyzed, 22 DAPs were located on chromosome A8. Four proteins among these, BnaA08T0019900WE (orthologous to eukaryotic translation initiation factor 2alpha kinase 1), BnaA08T0170800WE (heat shock 70 kDa protein 9), and BnaA08T0250400WE (mitochondrial intermembrane space import and assembly protein 40-like), and BnaA08T0284200WE (ran-binding protein 1 homolog a-like) showed shared pattern of protein accumulation where each protein increased in abundance in the CR-lines of both parents and the progeny (Supplementary Table 14D).

### Proteins Associated With Clubroot Resistance Related Genes and Quantitative Trait Loci

**FIGURE 9:**
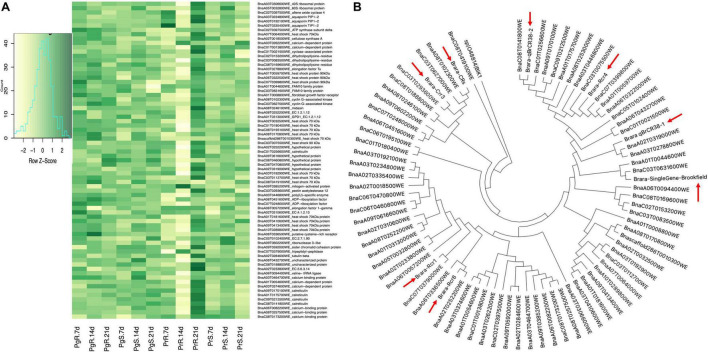
Differentially accumulated proteins (DAP) between inoculated- CR/CS progeny and CR/CS parent, these proteins are orthologous to clubroot resistance related proteins. QTL information (Rcr1, Rcr4, Rcr8, Crr3, CRs, SingleGene-Brookfield, and qBrCR38-2) was obtained from published articles (Hirai et al., 2004; Saito et al., 2006; Chu et al., 2014; Hasan and Rahman, 2016; Yu et al., 2017; Laila et al., 2019; Zhu et al., 2019). The physical location in each chromosome and reference sequence was based on *Brassica rapa* ‘Chifu-401’ whole genome sequence assembly V3.0 (http://brassicadb.cn/#/syntenic-gene/). **(A)** Heatmap shows the protein abundance of each CR related proteins at each time point across all genotypes investigated. **(B)** Dendrogram shows the distribution of putative proteins and the known clubroot resistance related proteins. The red solid arrows indicate the clubroot resistance loci. PgR-7d, CR progeny 7 DPI; PgR-14d, CR progeny 14 DPI; PgR-21d, CR progeny 21 DPI; PgS, CS progeny; PrR, CR parent; PrS, CS parent.

Our current study has identified 74 DAPs (Figure 9A) that are orthologous to the eight CR related loci (Rcr8, Crr3, Rcr4, Rcr1, CRs, SingleGene-Brookfield, and qBrCR38-1, qBrCR38-1) (Figure 9B) that were reported in the earlier studies (Hasan et al., 2021). The physical location of the DAPs in each chromosome was based on *Brassica rapa* ‘Chifu-401’ whole genome sequence assembly V3.0^1^. There were 57 unique proteins potentially involved in plant and pathogen interaction, orthologs of calcium binding-, heat shock-, and aquaporin- related proteins were frequently observed in the dataset (Supplementary Table 15). There were two orthologs of calcium binding proteins on chromosome A3, BnaA03T0464700WE (chromosome location, A3_24574673_25715499_Rcr1) and BnaA03T0374600WE (chromosome location, A3_23823130_26806601_Rcr4) that were increased in both the CR-progeny as well as CR-parent lines and decreased in both the CS-progeny and CS-parent lines (Supplementary Table 15). Similarly, among the heat shock proteins (HSP), Hsp70 and Hsp90 were predominant and BnaA03T0410600WE (chromosome location A8_20254038_21748038_qBrCR38-2) orthologous to Hsp70, consistently increased in both CR-parent and CR-progeny lines. Other putative proteins, BnaA09T0616600WE (chromosome location A7_20107291_20209491_qBrCR38-1) orthologous to D-mannose binding lectin, was increased in CR-progeny and CR-parent lines. Notably, BnaA06T0336500WE (chromosome location A3_23823130_26806601_Rcr4), orthologous to cysteine-rich receptor-like protein kinase), exhibited increasing pattern of protein abundance in the CR and CS lines in both the progeny and parent upon pathogen infection (Figure 9A). Finally, three putative orthologs of aquaporin proteins [BnaA03T0234800WE (chromosome location A3_23823130_26806601_Rcr4), BnaA03T0192100WE (chromosome location A8_12116024_13046214_SingleGene-Brookfield), and BnaA02T0335400WE (chromosome location A2_22502031_26348249_Rcr8)] were decreased in both CR- and CS-progeny lines upon pathogen infection. One of the proteins (BnaA02T0335400WE) was consistently decreased at all time points in both the CS and CR lines in the case of both the parent and progeny lines (Figure 9A).

## Discussion

Our current study has provided novel insights into the possible mechanisms underlying resistance to the clubroot pathogen. Although there are limited reports of proteomics and transcriptomics based investigation into the clubroot pathogen and *B. napus* interaction (Cao et al., 2008; Summanwar et al., 2019, 2020, 2021; Galindo-González et al., 2020; Zhou et al., 2020), our results align with earlier reports on *B. rapa* especially as it relates to calcium signaling protein kinases and calcium binding proteins in CR and CS root proteomes (Song et al., 2016). Similarly, the accumulation patterns of some of the vital defense-related glycosyl hydrolases including chitinases and thaumatin proteins, and phytohormone (e.g., SA)-related proteins were consistently increased in the CR root proteome which is in agreement with previous transcriptome studies (Summanwar et al., 2019; Galindo-González et al., 2020). Moreover, in this study we have identified several other calcium signaling proteins, dehydrins, and proteins that were orthologous to CR loci in different chromosomes particularly, chromosome A3 and A8. The DAPs that may be related to clubroot resistance are discussed in detail below.

### Calcium Signaling in Resistance to *Plasmodiophora brassicae*

When plants are challenged by pathogens, activation of multilayered intra- and extracellular signaling pathways takes place and defense responses are triggered (Tuteja and Mahajan, 2007). Calcium (Ca^2+^) ion concentration alters rapidly and dynamically in the cytosol in response to environmental or pathogen stimuli and plays a vital role as a second messenger, and communicates with other signal transduction pathways to elicit cellular responses (Zhang et al., 2014). Our current study identified 33 DAPs that are orthologous to various intracellular Ca^2+^ sensors, such as calreticulin, annexin, calmodulin (CaM), calmodulin-like (CMLs), and Ca^2+^ dependent protein kinases (Figure 7 and Supplementary Table 8A). We observed that the orthologs of sensor relay (CMLs) proteins (e.g., BnaA03T0464700WE, BnaC04T0461900WE, BnaC07T0227600WE, and BnaC08T0316200WE) consistently increased in the CR-progeny lines in response to the pathogen. This is similar to CMLs gene expression in *Arabidopsis* in response to *Pseudomonas syringae*, where the ortholog was rapidly induced under pathogen infection and its overexpression resulted in the hypersensitive response (Chiasson et al., 2005). Similarly, calcium dependent protein kinase (CPK; BnaA03T0374600WE) was increased in CR- parent and progeny lines and decreased in CS- parent and progeny indicating that the increase in accumulation in the CR lines could potentially be involved in the resistance phenotype. Similar observations have been made, albeit at the transcript level, where the CPK transcript was rapidly induced in response to fungal elicitors and its overexpression resulted in resistance to the pathogen (Coca and San Segundo, 2010).

In support of a potential role for calcium signaling proteins in mediating responses to *P. brassicae*, GO functional categories of response to - biotic (GO:0009607), and calcium ion binding (GO:0005509) were highly enriched. GO terms such as calcium ion binding (GO:0005509) is involved in the regulation of Ca^2+^ concentration in cytosol and Ca^2+^ sensor activity (Priest et al., 2014). Eight DAPs were associated with the term (GO:0005509) exhibiting a shared pattern between the CR-parent and progeny lines, with an increase in protein abundance as the infection progressed to 21 DPI (Supplementary Table 8B). Thus, the accumulation of calcium signaling-related proteins was strongly affected by *P. brassicae*, suggesting their involvement in mediating defense response against the pathogen.

### Reactive Oxygen Species in Response to *Plasmodiophora brassicae*

While excessive production of ROS in a cell can be detrimental, small and sub-toxic levels of ROS generation is essential for signaling purposes in order to maintain cellular processes, including plant-pathogen responses (Huang et al., 2019). Putative proteins related to diverse ROS-scavenging systems, such as peroxidases, ascorbate peroxidase, superoxide dismutase, glutathione, and thioredoxin have been reported to be differentially accumulated, potentially to maintain ROS homeostasis in cells and to limit the ROS-dependent damage (Mittler et al., 2004; Liu J. et al., 2021). Furthermore, increase in accumulation of some of the proteins, such as BnaA05T0420500WE (orthologous to peroxiredoxin-2F) and BnaA10T0260700WE (orthologous to l-ascorbate peroxidase 5) in the CR-progeny and a corresponding decrease in the CS-progeny, related to ROS could also suggest the involvement of ROS-dependent signaling pathways in clubroot resistance. For instance, GO enriched categories such as response to oxidative stress (GO:0006979), oxidoreductase (GO:0016491), antioxidant activity (GO:0016209), and acting on peroxide as acceptor (GO:0016684) were some of the highly enriched categories observed in both the genotypes. Furthermore, it has also been reported that ROS generation is linked to the formation of defensive barriers against biotrophic fungus in barley and inhibiting the pathogen colonization (Hückelhoven and Kogel, 2003). While ROS production in the cell was associated with the defense responses, some necrotrophic pathogens such as *Botrytis cinerea* promote ROS accumulation and triggers cell death aiding in the proliferation of the pathogen (Govrin and Levine, 2000). Earlier studies have demonstrated that overexpression of ROS related gene, l-ascorbate peroxidase 6, in *Nicotiana benthamiana* improved resistance against *Pseudomonas solanacearum* and *Fusarium solani* var. coeruleum in sugarcane (Liu et al., 2018). Similarly, the overexpression of ascorbate peroxidase lines in rice exhibited increased tolerance to *Xanthomonas oryzae* (Jiang et al., 2016). In both the cases, the genes were associated with ROS scavenging. However, to our knowledge, there are no reports of similar studies in the *Brassica-P. brassicae* pathosystem. Collectively, our results imply that the differential accumulation of ROS-related proteins may be the result of a complex network of signals against the pathogen attack and may be viable targets for genetic manipulation in order to afford durable resistance to *P. brassicae* in canola.

### Phytohormones and Immunity Responses to *Plasmodiophora brassicae*

Phytohormones play a vital role in responding to environmental stresses. In our study, proteins related to several phytohormones were detected including auxin, CKs, ET, JA, and SA. There are many reports of changes to endogenous phytohormone levels in plant roots during the development of clubroot infection (Ludwig-Müller et al., 2009). Primarily, SA and JA/ET are regulated synchronously to respond to the invading pathogen (Verma et al., 2016). SA plays a fundamental role in activating both systemic and local resistance in response to biotrophic pathogens (White, 1979; Vlot et al., 2009) while JA/ET is vital for defense against necrotrophic invaders (Glazebrook, 2005). Additionally, auxin and CKs, which are primarily involved in the regulation of cell division and cell elongation, are manipulated by *P. brassicae* leading to the hypertrophy of infected root cells (Robin et al., 2020). Our current study identified three auxin related proteins (BnaA10T0208100WE, BnaA05T0409700WE, and BnaC03T0302500WE) in which the putative protein orthologous to auxin efflux carrier component 5 (BnaA10T0208100WE) increased in the CR progeny and decreased in the CS progeny root proteomes as the infection progressed to 21 DPI (Figure 7). There are reports in the literature that the genes related to auxin efflux carrier was upregulated in the later stages of clubroot infection at 14-, 28-, and 35-DPI (Robin et al., 2020). In our study, at 21 DPI, one putative protein, BnaA06T0322500WE, orthologous to histidine kinase (Nishimura et al., 2004), was increased in the CR and decreased in the CS root proteomes of both parent and progeny lines (Supplementary Table 7B). Although it is unclear why these proteins are decreasing in the CS line as compared to CR line, it is a clear indication that they are important in mediating responses to clubroot pathogen. Additional studies are underway in our laboratory to further characterize the roles of these proteins in clubroot response/resistance.

NPR1 is one of the key positive regulators of SA dependent pathway and it has been demonstrated that it is important for the systemic acquired resistance in *Arabidopsis* (Cao et al., 1997). Overexpression of NPR1 in *B. juncea* conferred resistance against *Alternaria brassicae* and *Erysiphe cruciferarum* pathogen (Ali et al., 2017). Several studies in *Arabidopsis* and rice, have reported that accumulation of NPR1 conferred disease resistance against various pathogens (Fu and Dong, 2013; Kumar et al., 2013; Molla et al., 2016). Therefore, NPR1 related proteins exhibiting a contrasting pattern in abundance, especially those with an increase in CR root proteome and a decrease in CS, such as Bnascaffold286T0028200WE and BnaC07T0044900WE, may be potential candidates for further investigation into their role(s) in mediating resistance to clubroot.

Along with SA, ET, and JA also play crucial roles in the activation of plant defense pathways (Glazebrook, 2005). We have identified three proteins (BnaA03T0361400WE, BnaA09T0013600WE, and BnaC04T0098000WE) that are orthologous to *s*-adenosylmethionine (SAM) synthase, which increased in CR root proteomes of both parent and progeny lines in response to the pathogen. SAM is a precursor for the synthesis of ET, and the overexpression of SAM synthase related gene resulted in enhanced tolerance of Arabidopsis to oxidative stress (Ma et al., 2017). Moreover, earlier studies have demonstrated that, transcripts related to ET biosynthesis were induced in response to biotic stress (Vilanova et al., 2017; Yang et al., 2017). These proteins, especially the ones that increased in CR root proteomes and decreased in the CS may be appropriate candidates for further investigation.

### Dehydration Induced Proteins in Response to *Plasmodiophora brassicae*

Dehydrins are stress proteins and accumulate during abiotic stress and protect cells from dehydration (Kiyosue et al., 1994; Kovacs et al., 2008; Liu et al., 2017). Although their mechanisms of action are poorly understood, the accumulation of dehydrins in plants have been reported during salinity, drought, and low temperature stresses (Kovacs et al., 2008; Hanin et al., 2011). In this study, several dehydrin proteins that were observed to be decreased (BnaC02T0286500WE, and BnaA07T0239200WE) in the CS lines and some proteins (BnaA07T0354800WE, BnaC06T0291300WE, and BnaC06T0439800WE) were increased in the CS root proteome in response to the pathogen (Figure 7 and Supplementary Table 11A). Earlier studies have suggested that the expression of dehydrins may confer tolerance to desiccation stress in plants by acting as chaperones, thereby stabilizing cellular membranes by sequestering electrolytes, ions, buffering water molecules, and lowering electrolyte leakage (Rorat, 2006; Tunnacliffe and Wise, 2007; Graether and Boddington, 2014).

Microscopic observation of the disease progression in this study, especially as the pathogen progresses to secondary plasmodia phase, confirmed severe tissue damage presumably due to uncontrolled cell division and accumulation of callus around the vascular cambium. The proliferation of vascular cambium results in a concomitant inhibition of xylem development (Malinowski et al., 2012). Due to suppression of xylogenesis, plants experience dehydration due to poor water and mineral conduction (Malinowski et al., 2012) as evidenced by the wilting and stunting of plants at later stages of disease. Infected plants may trigger the expression of dehydrin genes leading to increase in dehydrin abundance in order to cope with the dehydration stress. Similarly, three proteins (BnaA08T0045300WE, BnaA10T0060800WE, and BnaC02T0364000WE) orthologous to other drought related genes, including cysteine proteinase RD21a (responsive to desiccation 21) (Yamaguchi-Shinozaki et al., 1992) were identified and all three proteins were increased in CR and CS lines; of note, the protein accumulation was relatively higher in the CS lines than in the CR lines (Supplementary Table 11A) supporting the hypothesis that clubroot infection results in a water-deficit stress in canola. To our knowledge, this is the first report of dehydrins being regulated in response to the clubroot pathogen. As alluded to above, our current data suggests that clubroot infection results in water deficit stress in canola plants mimicking drought stress as the xylogenesis is strongly repressed and uptake of water and mineral is obstructed in the CS lines. Additionally, it is reasonable to speculate that the increase in abundance of specific dehydrins (BnaC02T0286500WE and BnaA07T0239200WE) in the CR lines may be a contributing factor for the observed resistance in these lines and these proteins may be appropriate candidates for further investigation.

### Lignin Related Proteins in Plant Defense

Lignin, a phenolic polymer, forms a physical barrier between plant cell and pathogens and it is non-degradable by most microorganisms (Moura et al., 2010). Often a reinforcement of cell walls with lignin occurs when host encounters microbial pathogens (Moura et al., 2010; Vanholme et al., 2010). In this study, we identified 24 putative proteins that were orthologous to different types of peroxidases and proteins involved in lignin biosynthesis (Figure 7). For instance, the enzyme METK3 is involved in lignin biosynthesis (Shen et al., 2002) and there were four putative orthologs (BnaC03T0264500WE, BnaA09T0013600WE, BnaA 03T0361400WE, and BnaC04T0098000WE), of which, BnaA03T0361400WE (located in Chromosome A3) was increased consistently in the CR and decreased in CS lines especially when the infection progressed to 21 DPI. Similarly, one of the proteins orthologous to caffeic acid 3-*O*-methyltransferase (BnaA03T0142300WE, located at chromosome A3 genome) demonstrated shared pattern in protein accumulation in CR and CS lines in the case of both parent and progeny (Supplementary Table 9A). Similar to our results Zhang et al. (2007) reported an increase in peroxidase activity in Chinese cabbage in response to *Erwinia carotovora*. Increase in lignification has also been observed in *Pinus nigra* in response to *Sphaeropsis sapinea* infection (Bonello and Blodgett, 2003). There are also multiple reports in the literature describing an increase in the expression of lignin biosynthetic genes and lignin levels upon pathogen infection (Bhuiyan et al., 2009; Miedes et al., 2014; Summanwar et al., 2021). Over expression of lignin biosynthesis related genes such as BnaC03T0264500WE could potentially deposit lignin that could form a non-degradable barrier, which could restrict pathogen penetration of plant cells, thereby reducing disease infection. Others have reported that the overexpression of cinnamoyl-coA reductase 2, a rate limiting enzyme in lignin synthesis pathway, in *B. napus* resulted in increased lignin accumulation and a reduction in *Sclerotinia scletotiorum* disease symptoms (Liu D. et al., 2021). A similar approach could be viable for the development of CR canola.

### Clubroot Resistant Related Loci in Chromosome A3 and A8

Clubroot resistance-related genes have been reported on both A and C genomes of *Brassica* spp. A total of 24 CR loci have been reported, where 11 and five loci were reported from the chromosome A3 and A8, respectively; and the remaining were associated with other chromosomes (Hasan et al., 2021). In this study, we have identified 73 putative proteins from eight possible CR loci in Brassica that were reported earlier (Hirai et al., 2004; Saito et al., 2006; Chu et al., 2014; Hasan and Rahman, 2016; Yu et al., 2017; Laila et al., 2019; Zhu et al., 2019). Six proteins (BnaA02T0335400WE – related to Rcr8; BnaA06T0336500WE-, BnaC06T0195100WE-, BnaA03T0374600WE-, BnaA03T0262200WE- related to Rcr4, and BnaA03T0464700WE- related to Rcr1) increased in abundance and are potentially involved in plant and microbe interactions (Supplementary Table 8). The latter three (BnaA03T0374600WE, BnaA03T0262200WE, and BnaA03T0464700WE) are involved in calcium signaling, and the first three BnaA02T0335400WE, BnaA06T0336500WE, and BnaC06T0195100WE are related to aquaporin TIP1, cysteine rich receptor, and HSP70 protein, respectively (Supplementary Table 15). Putative proteins linked to Rcr4 are located at the chromosomal location between 23823130_26806601 bp in chromosome A3 of Brara_Chiifu_V3.0 genome, and the proteins linked to Rcr1 and Rcr8 are located at the chromosomal position 24574673_25715499 bp in A3 and 22502031_26348249 bp in A2, respectively. The locus labeled as Rcr4 on A03 was demonstrated to confer resistance against *P. brassicae* pathotypes 2, 3, 5, 6, and 8 (Yu et al., 2017). Likewise, the locus designated as Rcr8 on A2, and Rcr1 on A3 conferred resistance against pathotype 5X and 3, respectively (Chu et al., 2014; Yu et al., 2017). Many of the reported loci have been documented as major loci, QTL or genes, the putative proteins from our dataset were based on the sequence similarity with the specific loci and they will be suitable candidates for further investigation on their biological roles especially in the context of CR. They may also be suitable candidates for the design of gene-specific molecular markers to assist with the selection of CR lines.

## Conclusion

Our results have contributed to a better understanding of the protein level changes in *B. napus* in response to clubroot causing obligate biotrophic protist pathogen, *P. brassicae* and has identified major proteins and metabolic pathways potentially involved in clubroot resistance. Our results indicate that calcium signaling pathway is involved in intra- and extracellular flow of information during the pathogen detection and response against it and indicates that the accumulation of calcium ions enhances tolerance potential of host against biotic stress. Additionally, with the detection of important ROS related proteins, our results suggest that there could be a crosstalk between calcium and ROS in mediating a response to the clubroot pathogen. A range of metabolic pathways and host defense related proteins involving distinctive biological processes and molecular functions were either increased in the CR line indicating their potential role in the resistance mechanism or decreased in the CS line signifying the susceptible trait in the disease development. The former is supported by the higher abundance of lignin related proteins, ROS related peroxidases along with glycosyl hydrolases, PRRs, and phytohormone related proteins. This study has identified 74 differentially accumulated proteins that were linked to 7 possible clubroot resistance loci in four chromosomes (A2, A3, A7, and A8) and several of them were directly related to plant and pathogen interaction including calcium signaling and ROS related proteins. Many of the genes encoding the aforementioned proteins could be viable candidates for enhancing clubroot resistance in canola either through genetic manipulation or through classical breeding.

## Data Availability Statement

The datasets presented in this study can be found in online repositories. The names of the repository/repositories and accession number(s) can be found below: Proteomics raw data, Xcalibur parameters, and Spectronaut search settings have been deposited to the ProteomeXchange Consortium via the PRIDE partner repository with a dataset identifier PXD031070.

## Author Contributions

NK and HR designed the project with input from RGU on the proteomics component. NK supervised the project, interpreted the results, and edited multiple versions of the manuscript. DA designed, with the input of HR and NK, and conducted all the experiments, analyzed the results, and wrote the manuscript. DM and RGU performed the LC-MS/MS analysis and wrote the corresponding materials and methods. HR provided all plant material used in this study and input into draft version of this manuscript. All authors contributed to the article and approved the submitted version.

## Conflict of Interest

The authors declare that the research was conducted in the absence of any commercial or financial relationships that could be construed as a potential conflict of interest.

## Publisher’s Note

All claims expressed in this article are solely those of the authors and do not necessarily represent those of their affiliated organizations, or those of the publisher, the editors and the reviewers. Any product that may be evaluated in this article, or claim that may be made by its manufacturer, is not guaranteed or endorsed by the publisher.

## Footnotes

1
http://brassicadb.cn/#/syntenic-gene/

## References

[B1] Ali S. Mir Z. A. Tyagi A. Mehari H. Meena R. P. Bhat J. A. (2017). Overexpression of NPR1 in *Brassica junc*ea confers broad spectrum resistance to fungal pathogens. *Front. Plant Sci.* 8:1693. 10.3389/fpls.2017.01693 29046679 PMC5632730

[B2] Bhuiyan N. H. Selvaraj G. Wei Y. King J. (2009). Gene expression profiling and silencing reveal that monolignol biosynthesis plays a critical role in penetration defence in wheat against powdery mildew invasion. *J. Exp. Bot.* 60 509–521. 10.1093/jxb/ern290 19039100 PMC2651457

[B3] Bonello P. Blodgett J. T. (2003). *Pinus nigra*–*Sphaeropsis sapinea* as a model pathosystem to investigate local and systemic effects of fungal infection of pines. *Physiol. Mol. Plant Pathol.* 63 249–261. 10.1016/j.pmpp.2004.02.002

[B4] Buczacki S. T. (1983). “Plasmodiophora. an inter-relationship between biological and practical problems,” in *Zoosporic Plant Pathogens: A Modern Perspective*, ed. Buczacki S. T. (London: Academic Press Inc.), 161–191.

[B5] Canola Council of Canada (2021). *Industry Overview.* Available online at: https://www.canolacouncil.org/about-canola/industry/ (accessed December 27, 2021).

[B6] Cao H. Glazebrook J. Clarke J. D. Volko S. Dong X. (1997). The *Arabidopsis* NPR1 gene that controls systemic acquired resistance encodes a novel protein containing ankyrin repeats. *Cell* 88 57–63. 10.1016/S0092-8674(00)81858-9 9019406

[B7] Cao T. Srivastava S. Rahman M. H. Kav N. N. V. Hotte N. Deyholos M. K. (2008). Proteome-level changes in the roots of *Brassica napus* as a result of *Plasmodiophora brassicae* infection. *Plant Sci.* 174 97–115. 10.1016/j.plantsci.2007.10.002

[B8] Chen J. Pang W. Chen B. Zhang C. Piao Z. (2016). Transcriptome analysis of *Brassica rapa* near-isogenic lines carrying clubroot-resistant and –susceptible alleles in response to *Plasmodiophora brassicae* during early infection. *Front. Plant Sci.* 6:1183. 10.3389/fpls.2015.01183 26779217 PMC4700149

[B9] Chiasson D. Ekengren S. K. Martin G. B. Dobney S. L. Snedden W. A. (2005). Calmodulin-like proteins from *Arabidopsis* and tomato are involved in host defense against *Pseudomonas syringae* pv. tomato. *Plant Mol. Biol.* 58 887–897. 10.1007/s11103-005-8395-x 16240180

[B10] Chu M. Song T. Falk K. C. Zhang X. Liu X. Chang A. (2014). Fine mapping of Rcr1 and analyses of its effect on transcriptome patterns during infection by *Plasmodiophora brassicae*. *BMC Genom.* 15:1166. 10.1186/1471-2164-15-1166 25532522 PMC4326500

[B11] Coca M. San Segundo B. (2010). AtCPK1 calcium-dependent protein kinase mediates pathogen resistance in *Arabidopsis*: AtCPK1 is involved in pathogen resistance. *Plant J.* 63 526–540. 10.1111/j.1365-313X.2010.04255.x 20497373

[B12] Cook W. R. I. Schwartz E. J. (1930). The life-history, cytology and method of infection of *Plasmodiophora brassicae* woron. the cause of finger-and-toe disease of cabbages and other crucifers. *Philos. Trans. R. Soc. Lond. B Biol. Sci.* 218 283–314. 10.1098/rstb.1930.0006

[B13] Czubatka-Bieńkowska A. Kaczmarek J. Marzec-Schmidt K. Nieróbca A. Czajka A. Jêdryczka M. (2020). Country-wide qpcr based assessment of *Plasmodiophora brassicae* spread in agricultural soils and recommendations for the cultivation of Brassicaceae crops in poland. *Pathogens* 9:1070. 10.3390/pathogens9121070 33419297 PMC7766057

[B14] Dakouri A. Lamara M. Karim M. M. Wang J. Chen Q. Gossen B. D. (2021). Identification of resistance loci against new pathotypes of *Plasmodiophora brassicae* in *Brassica napus* based on genome-wide association mapping. *Sci. Rep.* 11:6599. 10.1038/s41598-021-85836-9 33758222 PMC7987998

[B15] Dixon G. R. (2009). The occurrence and economic impact of *Plasmodiophora brassicae* and clubroot disease. *J. Plant Growth Regul*. 28 194–202. 10.1007/s00344-009-9090-y

[B16] Donald C. Porter I. (2009). Integrated control of clubroot. *J. Plant Growth Regul.* 28 289–303. 10.1007/s00344-009-9094-7

[B17] Du Z. Zhou X. Ling Y. Zhang Z. Su Z. (2010). AgriGO: a GO analysis toolkit for the agricultural community. *Nucleic Acids Res.* 38 W64–W70. 10.1093/nar/gkq310 20435677 PMC2896167

[B18] Dunn O. J. (1961). multiple comparisons among means. *J. Am. Stat. Assoc.* 56 52–64. 10.1080/01621459.1961.10482090

[B19] Fu Z. Q. Dong X. (2013). Systemic acquired resistance: turning local infection into global defense. *Annu. Rev. Plant Biol.* 64 839–863. 10.1146/annurev-arplant-042811-105606 23373699

[B20] Galindo-González L. Manolii V. Hwang S.-F. Strelkov S. E. (2020). Response of *Brassica napus* to *Plasmodiophora brassicae* involves salicylic acid-mediated immunity: an rna-seq-based study. *Front. Plant Sci.* 11:1025. 10.3389/fpls.2020.01025 32754180 PMC7367028

[B21] Glazebrook J. (2005). Contrasting mechanisms of defense against biotrophic and necrotrophic pathogens. *Annu. Rev. Phytopathol.* 43 205–227. 10.1146/annurev.phyto.43.040204.135923 16078883

[B22] Gibbs J. G. (1931). Dissemination of clubroot in the dung from stock. *J. Agric.* 42 193–198.

[B23] Gossen B. D. Kasinathan H. Cao T. Manolii V. P. Strelkov S. E. Hwang S.-F. (2013). Interaction of pH and temperature affect infection and symptom development of *Plasmodiophora brassicae* in canola. *Can. J. Plant Pathol.* 35 294–303. 10.1080/07060661.2013.804882

[B24] Govrin E. M. Levine A. (2000). The hypersensitive response facilitates plant infection by the necrotrophic pathogen Botrytis cinerea. *Curr. Biol.* 10 751–757. 10.1016/S0960-9822(00)00560-1 10898976

[B25] Graether S. P. Boddington K. F. (2014). Disorder and function: a review of the dehydrin protein family. *Front. Plant Sci.* 5:576. 10.3389/fpls.2014.00576 25400646 PMC4215689

[B26] Hanin M. Brini F. Ebel C. Toda Y. Takeda S. Masmoudi K. (2011). Plant dehydrins and stress tolerance: versatile proteins for complex mechanisms. *Plant Signal. Behav.* 6 1503–1509. 10.4161/psb.6.10.17088 21897131 PMC3256378

[B27] Hasan J. Megha S. Rahman H. (2021). Clubroot in *Brassica*: recent advances in genomics, breeding, and disease management. *Genome* 64 735–760. 10.1139/gen-2020-0089 33651640

[B28] Hasan M. J. Rahman H. (2016). Genetics and molecular mapping of resistance to *Plasmodiophora brassicae* pathotypes 2, 3, 5, 6, and 8 in rutabaga (*Brassica napus* var. *napobrassica*). *Genome* 59 805–815. 10.1139/gen-2016-0034 27549861

[B29] Hirai M. Harada T. Kubo N. Tsukada M. Suwabe K. Matsumoto S. (2004). A novel locus for clubroot resistance in *Brassica rapa* and its linkage markers. *Theor. Appl. Genet.* 108 639–643. 10.1007/s00122-003-1475-x 14551685

[B30] Hollman K. B. Hwang S. F. Manolii V. P. Strelkov S. E. (2021). Pathotypes of *Plasmodiophora brassicae* collected from clubroot resistant canola (*Brassica napus* L.) cultivars in western Canada in 2017-2018. *Can. J. Plant Pathol.* 43 622–630. 10.1080/07060661.2020.1851893

[B31] Howard R. J. Strelkov S. E. Harding M. W. (2010). Clubroot of cruciferous crops – new perspectives on an old disease†. *Can. J. Plant Pathol.* 32 43–57. 10.1080/07060661003621761

[B32] Huang H. Ullah F. Zhou D.-X. Yi M. Zhao Y. (2019). Mechanisms of ROS regulation of plant development and stress responses. *Front. Plant Sci.* 10:800. 10.3389/fpls.2019.00800 31293607 PMC6603150

[B33] Hückelhoven R. Kogel K.-H. (2003). Reactive oxygen intermediates in plant-microbe interactions: who is who in powdery mildew resistance? *Planta* 216 891–902. 10.1007/s00425-003-0973-z 12687357

[B34] Hwang S.-F. Strelkov S. E. Feng J. Gossen B. D. Howard R. J. (2012). *Plasmodiophora brassicae*: a review of an emerging pathogen of the Canadian canola (*Brassica napus*) crop: progress on canola clubroot research. *Mol. Plant Pathol.* 13 105–113. 10.1111/j.1364-3703.2011.00729.x 21726396 PMC6638701

[B35] Jahn L. Mucha S. Bergmann S. Horn C. Staswick P. Steffens B. (2013). The clubroot pathogen (*plasmodiophora brassicae*) influences auxin signaling to regulate auxin homeostasis in *Arabidopsis*. *Plants* 2 726–749. 10.3390/plants2040726 27137401 PMC4844388

[B36] Ji R. Wang Y. Wang X. Liu Y. Shen X. Feng H. (2018). Proteomic analysis of the interaction between *Plasmodiophora brassicae* and Chinese cabbage (*Brassica rapa* L. ssp. *pekinensis*) at the initial infection stage. *Sci. Hortic.* 233 386–393. 10.1016/j.scienta.2018.02.006

[B37] Jiang G. Yin D. Zhao J. Chen H. Guo L. Zhu L. (2016). The rice thylakoid membrane-bound ascorbate peroxidase OsAPX8 functions in tolerance to bacterial blight. *Sci. Rep.* 6:26104. 10.1038/srep26104 27185545 PMC4868969

[B38] Kageyama K. Asano T. (2009). Life cycle of *Plasmodiophora brassicae*. *J. Plant Growth Regul.* 28 203–211. 10.1007/s00344-009-9101-z

[B39] Kanehisa M. Sato Y. Kawashima M. Furumichi M. Tanabe M. (2016). KEGG as a reference resource for gene and protein annotation. *Nucleic Acids Res.* 44 D457–D462. 10.1093/nar/gkv1070 26476454 PMC4702792

[B40] Keen N. T. Williams P. H. (1969). Translocation of sugars into infected cabbage tissues during clubroot development. *Plant Physiol.* 44 748–754. 10.1104/pp.44.5.748 16657127 PMC396155

[B41] Kieber J. J. Rothenberg M. Roman G. Feldmann K. A. Ecker J. R. (1993). CTR1, a negative regulator of the ethylene response pathway in *Arabidopsis*, encodes a member of the Raf family of protein kinases. *Cell* 72 427–441. 10.1016/0092-8674(93)90119-B 8431946

[B42] Kiyosue T. Yamaguchi-Shinozaki K. Shinozaki K. (1994). Cloning of cDNAs for genes that are early-responsive to dehydration stress (ERDs) in *Arabidopsis thaliana* L.: identification of three ERDs as HSP cognate genes. *Plant Mol. Biol.* 25 791–798. 10.1007/BF00028874 8075396

[B43] Kovacs D. Kalmar E. Torok Z. Tompa P. (2008). Chaperone activity of ERD10 and ERD14, two disordered stress-related plant proteins. *Plant Physiol.* 147 381–390. 10.1104/pp.108.118208 18359842 PMC2330285

[B44] Kuginuki Y. Yoshikawa H. Hirai M. (1999). Variation in virulence of *Plasmodiophora brassicae* in Japan tested with clubroot-resistant cultivars of Chinese cabbage (*Brassica rapa* L. ssp. *pekinensis*). *Eur. J. Plant Pathol.* 6 327–332.

[B45] Kumar V. Joshi S. G. Bell A. A. Rathore K. S. (2013). Enhanced resistance against *Thielaviopsis basicola* in transgenic cotton plants expressing *Arabidopsis* NPR1 gene. *Transgenic Res.* 22 359–368. 10.1007/s11248-012-9652-9 23001518

[B46] Laila R. Park J.-I. Robin A. H. K. Natarajan S. Vijayakumar H. Shirasawa K. (2019). Mapping of a novel clubroot resistance QTL using ddRAD-seq in Chinese cabbage (*Brassica rapa* L.). *BMC Plant Biol.* 19:13. 10.1186/s12870-018-1615-8 30621588 PMC6325862

[B47] Lan M. Li G. Hu J. Yang H. Zhang L. Xu X. (2019). iTRAQ-based quantitative analysis reveals proteomic changes in Chinese cabbage (*Brassica rapa* L.) in response to *Plasmodiophora brassicae* infection. *Sci. Rep.* 9:12058. 10.1038/s41598-019-48608-0 31427711 PMC6700187

[B48] Leutert M. Rodríguez-Mias R. A. Fukuda N. K. Villén J. (2019). R2-P2 rapid-robotic phosphoproteomics enables multidimensional cell signaling studies. *Mol. Syst. Biol.* 15:e9021. 10.15252/msb.20199021 31885202 PMC6920700

[B49] Li L. Luo Y. Chen B. Xu K. Zhang F. Li H. (2016). A genome-wide association study reveals new loci for resistance to clubroot disease in *Brassica napus*. *Front. Plant Sci.* 7:1483. 10.3389/fpls.2016.01483 27746804 PMC5044777

[B50] Liu D. Wu J. Lin L. Li P. Li S. Wang Y. (2021). Overexpression of cinnamoyl-coa reductase 2 in brassica napus increases resistance to *Sclerotinia sclerotiorum* by affecting lignin biosynthesis. *Front. Plant Sci.* 12:732733. 10.3389/fpls.2021.732733 34630482 PMC8494948

[B51] Liu F. Huang N. Wang L. Ling H. Sun T. Ahmad W. (2018). A novel l-ascorbate peroxidase 6 gene, scapx6, plays an important role in the regulation of response to biotic and abiotic stresses in sugarcane. *Front. Plant Sci.* 8:2262. 10.3389/fpls.2017.02262 29387074 PMC5776131

[B52] Liu J. Fu C. Li G. Khan M. N. Wu H. (2021). ROS homeostasis and plant salt tolerance: plant nanobiotechnology updates. *Sustainability* 13:3552. 10.3390/su13063552

[B53] Liu Y. Song Q. Li D. Yang X. Li D. (2017). Multifunctional roles of plant dehydrins in response to environmental stresses. *Front. Plant Sci.* 8:1018. 10.3389/fpls.2017.01018 28649262 PMC5465263

[B54] Ludwig-Müller J. Auer S. Jülke S. Marschollek S. (2017). “Manipulation of auxin and cytokinin balance during the *Plasmodiophora brassicae–Arabidopsis thaliana* interaction, in *auxins and cytokinins*,” in *Plant Biology* Methods in Molecular Biology, eds Dandekar T. Naseem M. (New York, NY: Springer), 41–60. 10.1007/978-1-4939-6831-2_3 28265986

[B55] Ludwig-Müller J. Prinsen E. Rolfe S. A. Scholes J. D. (2009). Metabolism and plant hormone action during clubroot disease. *J. Plant Growth Regul.* 28 229–244. 10.1007/s00344-009-9089-4

[B56] Ma C. Wang Y. Gu D. Nan J. Chen S. Li H. (2017). Overexpression of s-adenosyl-l-methionine synthetase 2 from sugar beet m14 increased arabidopsis tolerance to salt and oxidative stress. *IJMS* 18:847. 10.3390/ijms18040847 28420190 PMC5412431

[B57] Malinowski R. Smith J. A. Fleming A. J. Scholes J. D. Rolfe S. A. (2012). Gall formation in clubroot-infected *Arabidopsis* results from an increase in existing meristematic activities of the host but is not essential for the completion of the pathogen life cycle: gall formation in clubroot infection. *Plant J.* 71 226–238. 10.1111/j.1365-313X.2012.04983.x 22394393

[B58] Mehta D. Scandola S. Uhrig G. R. (2022). BoxCar and library free data independent acquisition substantially improve the depth, range, and completeness of label-free quantitative proteomics. *Anal. Chem.* 94 793–802. 10.1021/acs.analchem.1c03338 34978796

[B59] Miedes E. Vanholme R. Boerjan W. Molina A. (2014). The role of the secondary cell wall in plant resistance to pathogens. *Front. Plant Sci.* 5:358. 10.3389/fpls.2014.00358 25161657 PMC4122179

[B60] Mistry J. Chuguransky S. Williams L. Qureshi M. Salazar G. A. Sonnhammer E. L. L. (2021). Pfam: the protein families database in 2021. *Nucleic Acids Res.* 49 D412–D419. 10.1093/nar/gkaa913 33125078 PMC7779014

[B61] Mittler R. Vanderauwera S. Gollery M. Van Breusegem F. (2004). Reactive oxygen gene network of plants. *Trends Plant Sci.* 9 490–498. 10.1016/j.tplants.2004.08.009 15465684

[B62] Molla K. A. Karmakar S. Chanda P. K. Sarkar S. N. Datta S. K. Datta K. (2016). Tissue-specific expression of *Arabidopsis* NPR1 gene in rice for sheath blight resistance without compromising phenotypic cost. *Plant Sci.* 250 105–114. 10.1016/j.plantsci.2016.06.005 27457988

[B63] Moura J. C. M. S. Bonine C. A. V. de Oliveira Fernandes Viana J. Dornelas M. C. Mazzafera P. (2010). Abiotic and biotic stresses and changes in the lignin content and composition in plants. *J. Integr. Plant Biol.* 52 360–376. 10.1111/j.1744-7909.2010.00892.x 20377698

[B64] Nagaoka T. Doullah M. A. U. Matsumoto S. Kawasaki S. Ishikawa T. Hori H. (2010). Identification of QTLs that control clubroot resistance in Brassica oleracea and comparative analysis of clubroot resistance genes between B. rapa and B. oleracea. *Theor. Appl. Genet.* 120 1335–1346. 10.1007/s00122-010-1259-z 20069415

[B65] Nishimura C. Ohashi Y. Sato S. Kato T. Tabata S. Ueguchi C. (2004). Histidine kinase homologs that act as cytokinin receptors possess overlapping functions in the regulation of shoot and root growth in arabidopsis. *Plant Cell* 16 1365–1377. 10.1105/tpc.021477 15155880 PMC490032

[B66] Pedras M. S. C. Zheng Q.-A. Strelkov S. (2008). Metabolic changes in roots of the oilseed canola infected with the biotroph *Plasmodiophora brassicae:* phytoalexins and phytoanticipins. *J. Agric. Food Chem.* 56 9949–9961. 10.1021/jf802192f 18834132

[B67] Priest H. D. Fox S. E. Rowley E. R. Murray J. R. Michael T. P. Mockler T. C. (2014). Analysis of global gene expression in *Brachypodium distachyon* reveals extensive network plasticity in response to abiotic stress. *PLoS One* 9:e87499. 10.1371/journal.pone.0087499 24489928 PMC3906199

[B68] Quirino B. F. Candido E. S. Campos P. F. Franco O. L. Krüger R. H. (2010). Proteomic approaches to study plant–pathogen interactions. *Phytochemistry* 71 351–362. 10.1016/j.phytochem.2009.11.005 20005547

[B69] Rahman H. (2016). UA AlfaGold clearfield herbicide-tolerant spring *Brassica napus* canola developed from winter × spring canola cross. *Can. J. Plant Sci.* 97 144–146. 10.1139/CJPS-2016-0028

[B70] Robin A. H. K. Saha G. Laila R. Park J.-I. Kim H.-T. Nou I.-S. (2020). Expression and role of biosynthetic, transporter, receptor, and responsive genes for auxin signaling during clubroot disease development. *IJMS* 21:5554. 10.3390/ijms21155554 32756478 PMC7432499

[B71] Rorat T. (2006). Plant dehydrins — Tissue location, structure and function. *Cell. Mol. Biol. Lett.* 11 536–556. 10.2478/s11658-006-0044-0 16983453 PMC6275985

[B72] Saito M. Kubo N. Matsumoto S. Suwabe K. Tsukada M. Hirai M. (2006). Fine mapping of the clubroot resistance gene, Crr3, in *Brassica rapa*. *Theor. Appl. Genet.* 114 81–91. 10.1007/s00122-006-0412-1 17039346

[B73] Shahbandeh M. (2021). *Global Oilseed Production in 2020/2021, by type: Statista 2021.* Available online at: https://www.statista.com/statistics/267271/worldwide-oilseed-production-since-2008/ (accessed December 27, 2021).

[B74] Shen B. Li C. Tarczynski M. C. (2002). High free-methionine and decreased lignin content result from a mutation in the *Arabidopsis S* -adenosyl-L-methionine synthetase 3 gene. *Plant J.* 29 371–380. 10.1046/j.1365-313X.2002.01221.x 11844113

[B75] Siemens J. Keller I. Sarx J. Kunz S. Schuller A. Nagel W. (2006). Transcriptome analysis of *Arabidopsis* clubroots indicate a key role for cytokinins in disease development. *MPMI* 19 480–494. 10.1094/MPMI-19-0480 16673935

[B76] Song J. M. Guan Z. Hu J. Guo C. Yang Z. Wang S. (2020). Eight high-quality genomes reveal pan-genome architecture and ecotype differentiation of *Brassica napus*. *Nat. Plants* 6 34–45. 10.1038/s41477-019-0577-7 31932676 PMC6965005

[B77] Song T. Chu M. Lahlali R. Yu F. Peng G. (2016). Shotgun label-free proteomic analysis of clubroot (*Plasmodiophora brassicae*) resistance conferred by the gene Rcr1 in *Brassica rapa*. *Front. Plant Sci.* 7:1013. 10.3389/fpls.2016.01013 27462338 PMC4939851

[B78] Strelkov S. E. Hwang S.-F. Manolii V. P. Cao T. Fredua-Agyeman R. Harding M. W. (2018). Virulence and pathotype classification of *Plasmodiophora brassicae* populations collected from clubroot resistant canola (*Brassica napus*) in Canada. *Can. J. Plant Pathol.* 40 284–298. 10.1080/07060661.2018.1459851

[B79] Su T. Yu S. Wang W. Li P. Zhang F. Yu Y. (2018). iTRAQ analysis of protein profile during the secondary stage of infection of *Plasmodiophora brassicae* in Chinese cabbage (*Brassica rapa* subsp. *pekinensis*). *J. Plant Pathol.* 100 533–542. 10.1007/s42161-018-0121-z

[B80] Summanwar A. Basu U. Kav N. N. V. Rahman H. (2021). Identification of lncRNAs in response to infection by *Plasmodiophora brassicae* in *Brassica napus* and development of lncRNA-based SSR markers. *Genome* 64 547–566. 10.1139/gen-2020-0062 33170735

[B81] Summanwar A. Basu U. Rahman H. Kav N. (2019). Identification of lncRNAs responsive to infection by *Plasmodiophora brassicae* in clubroot-susceptible and -resistant *Brassica napus* lines carrying resistance introgressed from Rutabaga. *MPMI.* 32 1360–1377. 10.1094/MPMI-12-18-0341-R 31090490

[B82] Summanwar A. Basu U. Rahman H. Kav N. N. V. (2020). Non-coding RNAs as emerging targets for crop improvement. *Plant Sci.* 297:110521. 10.1016/j.plantsci.2020.110521 32563460

[B83] Tian T. Liu Y. Yan H. You Q. Yi X. Du Z. (2017). agriGO v2.0: a GO analysis toolkit for the agricultural community, 2017 update. *Nucleic Acids Res.* 45 W122–W129. 10.1093/nar/gkx382 28472432 PMC5793732

[B84] Tunnacliffe A. Wise M. J. (2007). The continuing conundrum of the LEA proteins. *Naturwissenschaften* 94 791–812. 10.1007/s00114-007-0254-y 17479232

[B85] Tuteja N. Mahajan S. (2007). Calcium signaling network in plants: an overview. *Plant Signal. Behav.* 2 79–85. 10.4161/psb.2.2.4176 19516972 PMC2633903

[B86] Vanholme R. Demedts B. Morreel K. Ralph J. Boerjan W. (2010). Lignin biosynthesis and structure. *Plant Physiol.* 153 895–905. 10.1104/pp.110.155119 20472751 PMC2899938

[B87] Verma V. Ravindran P. Kumar P. P. (2016). Plant hormone-mediated regulation of stress responses. *BMC Plant Biol.* 16:86. 10.1186/s12870-016-0771-y 27079791 PMC4831116

[B88] Vilanova L. Vall-llaura N. Torres R. Usall J. Teixidó N. Larrigaudière C. (2017). *Penicillium expansum* (compatible) and *Penicillium digitatum* (non-host) pathogen infection differentially alter ethylene biosynthesis in apple fruit. *Plant Physiol. Biochem.* 120 132–143. 10.1016/j.plaphy.2017.09.024 29028545

[B89] Vlot A. C. Dempsey D. A. Klessig D. F. (2009). Salicylic acid, a multifaceted hormone to combat disease. *Annu. Rev. Phytopathol.* 47 177–206. 10.1146/annurev.phyto.050908.135202 19400653

[B90] Wagner G. Charton S. Lariagon C. Laperche A. Lugan R. Hopkins J. (2012). Metabotyping: a new approach to investigate rapeseed (*Brassica napus* L.) genetic diversity in the metabolic response to clubroot infection. *MPMI* 25 1478–1491. 10.1094/MPMI-02-12-0032-R 22809276

[B91] Wagner G. Laperche A. Lariagon C. Marnet N. Renault D. Guitton Y. (2019). Resolution of quantitative resistance to clubroot into QTL-specific metabolic modules. *J. Exp. Bot.* 70 5375–5390. 10.1093/jxb/erz265 31145785 PMC6793449

[B92] Walerowski P. Gündel A. Yahaya N. Truman W. Sobczak M. Olszak M. (2018). Clubroot disease stimulates early steps of phloem differentiation and recruits SWEET sucrose transporters within developing galls. *Plant Cell* 30 3058–3073. 10.1105/tpc.18.00283 30413655 PMC6354258

[B93] Wallenhammar A. -C. (1996). Prevalence of *Plasmodiophora brassicae* in a spring oilseed rape growing area in central Sweden and factors influencing soil infestation levels. *Plant Pathol.* 45 710–719. 10.1046/j.1365-3059.1996.d01-173.x

[B94] White R. F. (1979). Acetylsalicylic acid (aspirin) induces resistance to tobacco mosaic virus in tobacco. *Virology* 99 410–412. 10.1016/0042-6822(79)90019-9 18631626

[B95] Williams P. H. (1966). A system for the determination of races of *Plasmodiophorae brassicae* that infect cabbage and rutabaga. *Phytopathology* 56 624–626.

[B96] Yamaguchi-Shinozaki K. Koizumi M. Urao S. Shinozaki K. (1992). Molecular cloning and characterization of 9 cDNAs for genes that are responsive to desiccation in *Arabidopsis thaliana*: sequence analysis of one cDNA clone that encodes a putative transmembrane channel protein. *Plant Cell Physiol.* 33 217–224. 10.1093/oxfordjournals.pcp.a078243

[B97] Yang C. Li W. Cao J. Meng F. Yu Y. Huang J. (2017). Activation of ethylene signaling pathways enhances disease resistance by regulating ROS and phytoalexin production in rice. *Plant J.* 89 338–353. 10.1111/tpj.13388 27701783

[B98] Yu F. Zhang X. Peng G. Falk K. C. Strelkov S. E. Gossen B. D. (2017). Genotyping-by-sequencing reveals three QTL for clubroot resistance to six pathotypes of *Plasmodiophora brassicae* in *Brassica rapa*. *Sci. Rep.* 7:4516. 10.1038/s41598-017-04903-2 28674416 PMC5495781

[B99] Zhang L. Du L. Poovaiah B. W. (2014). Calcium signaling and biotic defense responses in plants. *Plant Signal. Behav.* 9:e973818. 10.4161/15592324.2014.973818 25482778 PMC4623097

[B100] Zhang S.-H. Yang Q. Ma R.-C. (2007). *Erwinia carotovora* ssp. *carotovora* infection induced defense lignin accumulation and lignin biosynthetic gene expression in Chinese Cabbage (*Brassica rapa* L. ssp. *pekinensis*). *J. Integr. Plant Biol.* 49 993–1002. 10.1111/j.1672-9072.2007.00478.x

[B101] Zhang X. Liu Y. Fang Z. Li Z. Yang L. Zhuang M. (2016). Comparative transcriptome analysis between Broccoli (*Brassica oleracea* var. italica) and wild cabbage (*Brassica macrocarpa* Guss.) in response to *Plasmodiophora brassicae* during different infection stages. *Front. Plant Sci.* 7:1929. 10.3389/fpls.2016.01929 28066482 PMC5179516

[B102] Zhao Y. Bi K. Gao Z. Chen T. Liu H. Xie J. (2017). Transcriptome analysis of *Arabidopsis thaliana* in response to *Plasmodiophora brassicae* during early infection. *Front. Microbiol.* 8:673. 10.3389/fmicb.2017.00673 28484434 PMC5401899

[B103] Zhou Q. Galindo-González L. Manolii V. Hwang S.-F. Strelkov S. E. (2020). Comparative transcriptome analysis of rutabaga (*Brassica napus*) cultivars indicates activation of salicylic acid and ethylene-mediated defenses in response to *Plasmodiophora brassicae*. *IJMS* 21:8381. 10.3390/ijms21218381 33171675 PMC7664628

[B104] Zhu H. Zhai W. Li X. Zhu Y. (2019). Two QTLs controlling clubroot resistance identified from bulked segregant sequencing in Pakchoi (*Brassica campestris* ssp. chinensis Makino). *Sci. Rep.* 9:9228. 10.1038/s41598-019-44724-z 31239512 PMC6592919

