# Corrigendum: A proteome-level investigation into *Plasmodiophora brassicae* resistance in *Brassica napus* canola

**DOI:** 10.3389/fpls.2025.1597953

**Published:** 2025-05-08

**Authors:** Dinesh Adhikary, Devang Mehta, R. Glen Uhrig, Habibur Rahman, Nat N. V. Kav

**Affiliations:** ^1^ Department of Agricultural, Food and Nutritional Science, University of Alberta, Edmonton, AB, Canada; ^2^ Department of Biological Sciences, University of Alberta, Edmonton, AB, Canada

**Keywords:** *Brassica napus*, clubroot, proteomics, calcium binding, plant–pathogen interaction

In the published article, there was an error in one of the panel figures under 7 DPI. The panel figures under 7 DPI were duplicated by mistake with our other published article in the same year (Adhikary et al., 2022; DOI: 10.1039/d2mo00251e). The corrected panel figure for 7 DPI in **Figures 1**–**3** are provided with this letter. The caption of the figures does not change, and other images on the panel stay the same as it was in the original article. Figure legends are shown below.

**Figure 1:**
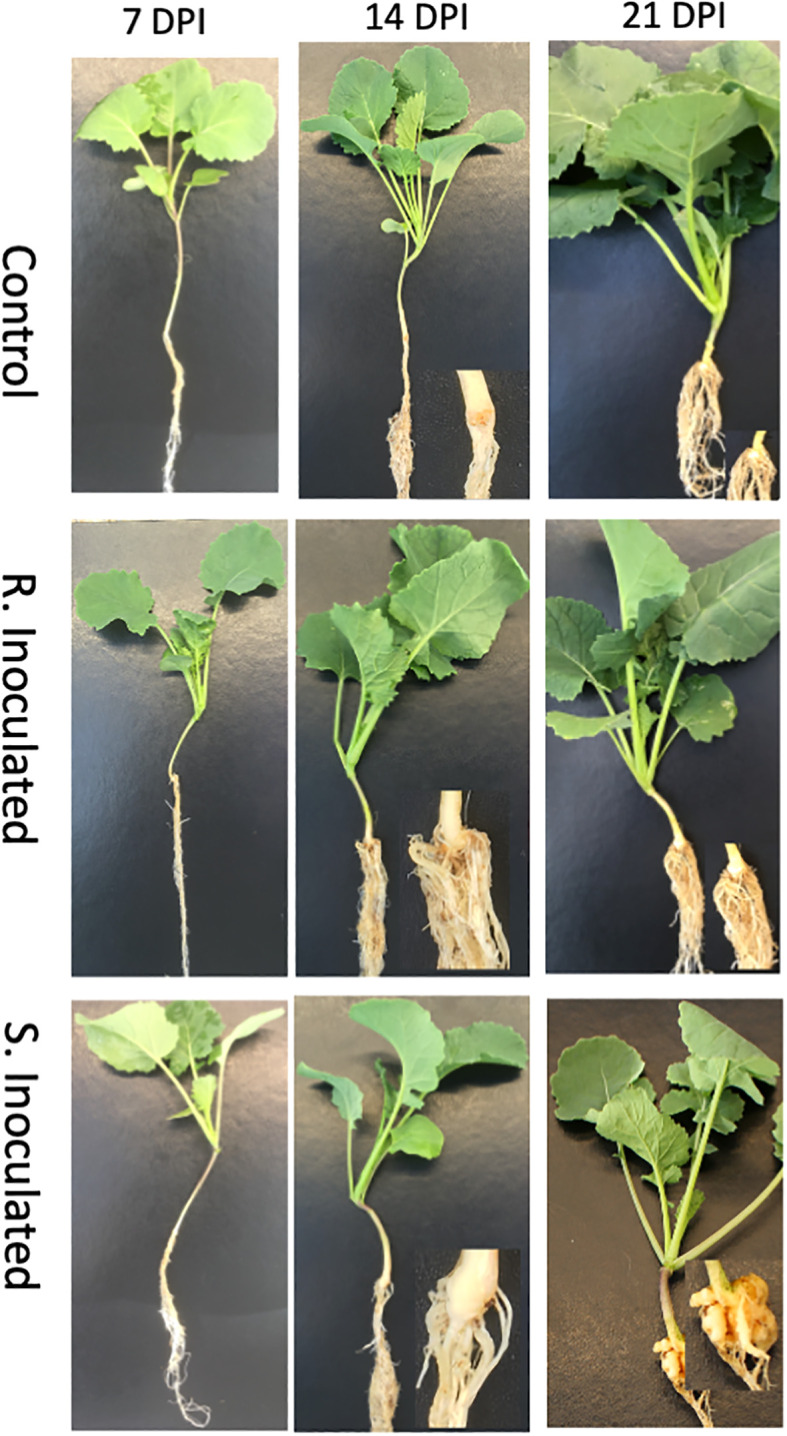
Clubroot gall development following inoculation with *P. brassicae* pathotype 3. Control at 7-, 14-, and 21-DPI, Clubroot resistant (CR) inoculated line at 7-, 14-, and 21-DPI Clubroot susceptible (CS) inoculated 7-, 14-, and 21-DPI.

**Figure 2:**
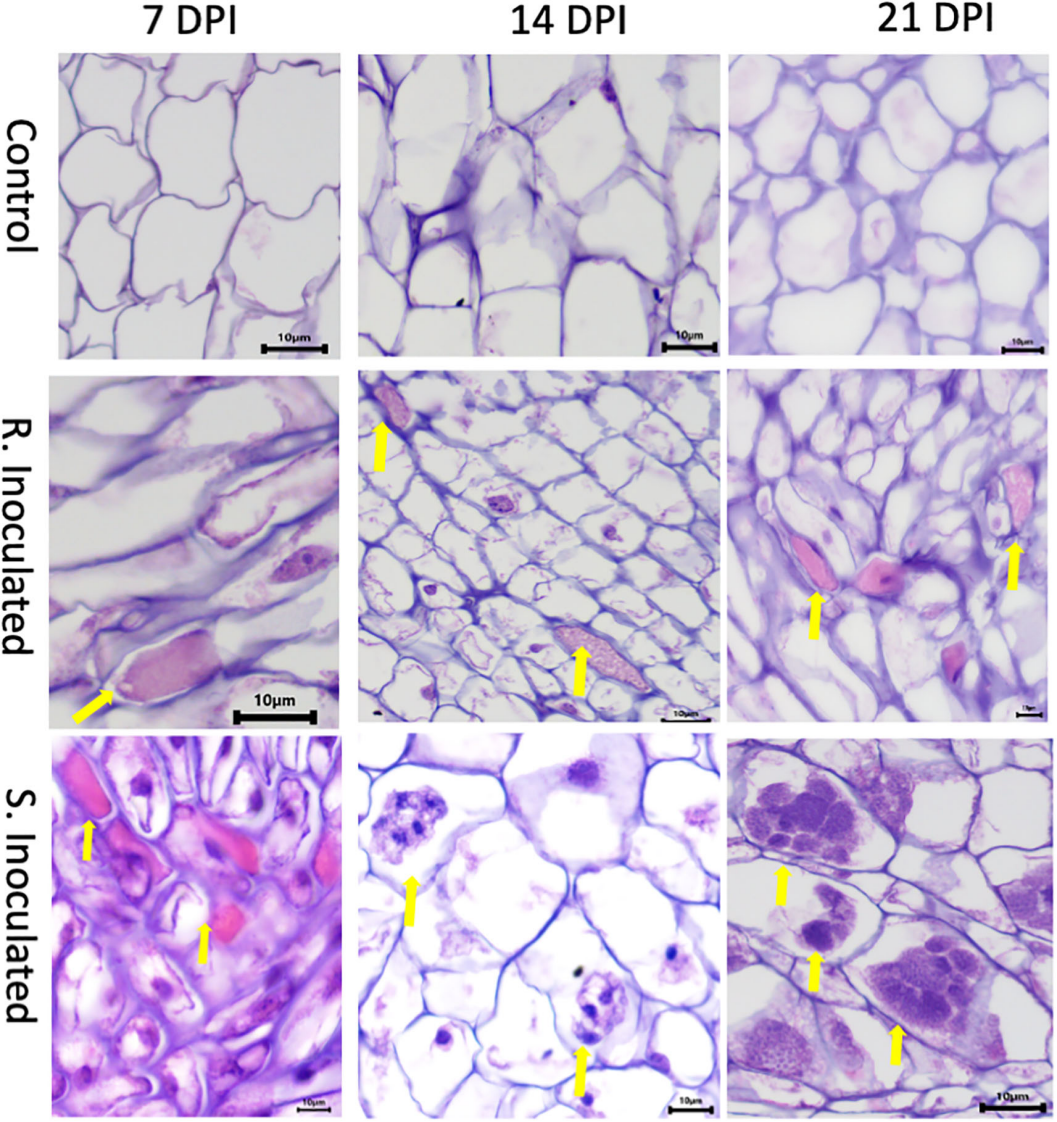
Histology images of root cross sections after *P. brassicae* infection at 7-, 14-, and 21-days post inoculation (DPI). Root tissues were stained with eosin and hematoxylin. Each column indicates DPI of the pathogen and the rows show the control and inoculated genotypes [clubroot- susceptible (CS) and resistant (CR) lines]. At 7 DPI, infected cells showed primary plasmodia with dark purple mass within cells indicated by the solid yellow arrow. At 14 DPI, CS inoculated line showed the presence of secondary plasmodia; however, the pathogen development on the CR inoculated line was not progressed to secondary plasmodia phase. At 21 DPI, pathogen clearly progressed to secondary plasmodia phase, maturing into developing resting spores in the CS line. However, the infection development was not progressed further at the same time point on the CR line.

**Figure 3:**
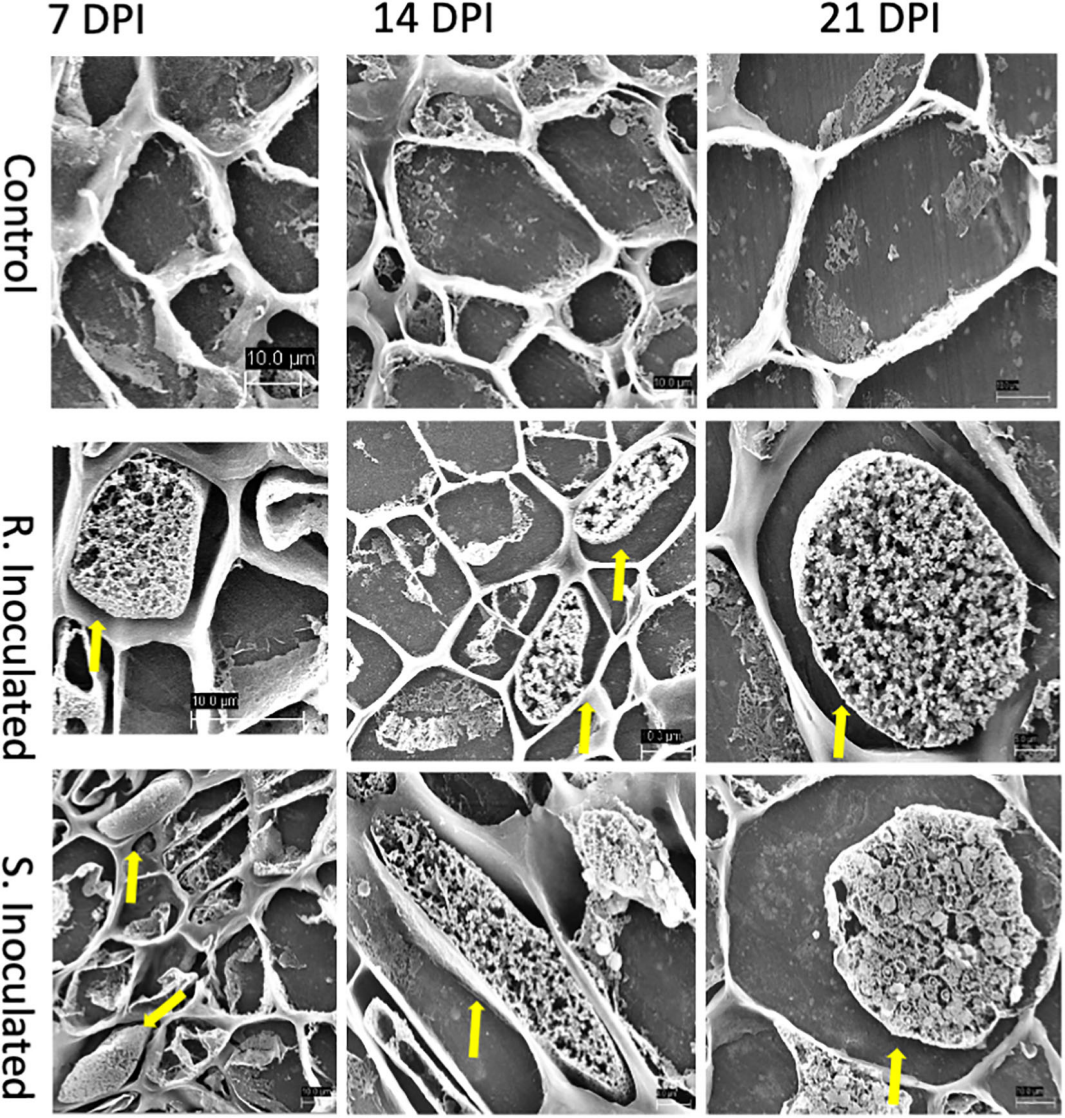
Scanning electron micrograph (SEM) of root cross sections after *P. brassicae* infection at 7-, 14-, and 21-DPI. Each column indicates days after inoculation of the pathogen and the rows show the control and inoculated genotypes (CR and CS). At 7 DPI, infected cells showed primary plasmodia within cells indicated by the solid yellow arrow. At 14 DPI, CS inoculated line showed the presence of secondary plasmodia; however, the pathogen development on the CR inoculated line was not progressed to secondary plasmodia phase. At 21 DPI, pathogen clearly progressed to secondary plasmodia phase, maturing into resting spores. However, the infection development was not progressed in the CR line at the timepoint.

We apologize for this error and state that this correction does not change the scientific conclusions of the article in any way.

