# Supplementary figures and images for: A Proteome-Level Investigation Into Plasmodiophora brassicae Resistance in Brassica napus Canola
Source: Front Plant Sci. 2022 Mar 24;13:860393. doi: 10.3389/fpls.2022.860393 (PMC8988049; doi:10.3389/fpls.2022.860393)

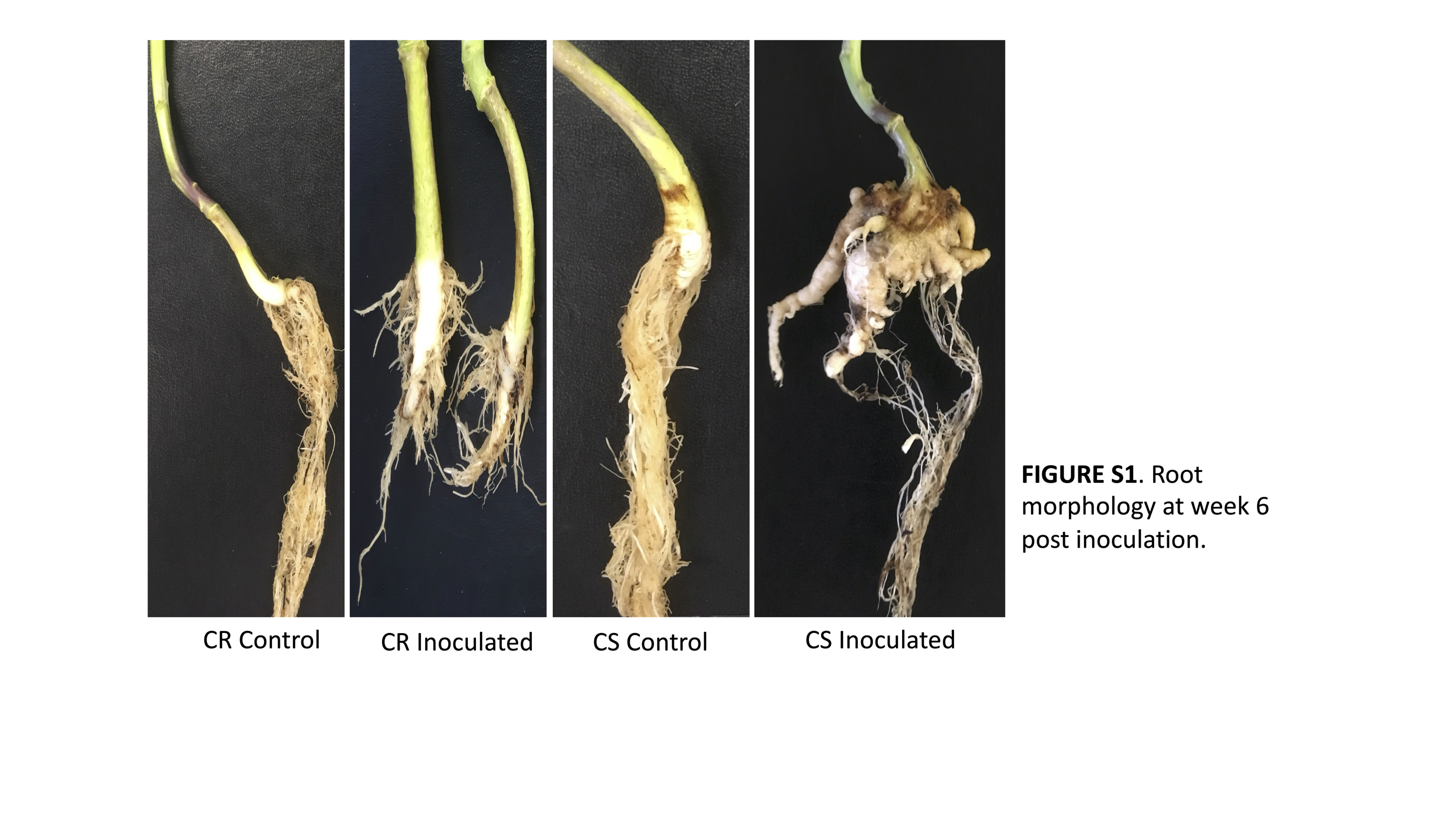

Supplement: Supplementary file 1 [file Image_1.JPEG]
